# Aqueous Solutions of Associating Poly(acrylamide-*co*-styrene): A Path to Improve Drag Reduction?

**DOI:** 10.1021/acs.macromol.2c01219

**Published:** 2022-11-30

**Authors:** Emina Muratspahić, Lukas Brandfellner, Jana Schöffmann, Alexander Bismarck, Hans Werner Müller

**Affiliations:** † Institute of Materials Chemistry and Research, Polymer and Composite Engineering (PaCE) Group, University of Vienna, Währinger Straße 42, 1090Vienna, Austria; ‡ Doctoral College Advanced Functional Materials, University of Vienna, Strudlhofgasse 4, 1090Vienna, Austria; § Department of Chemical Engineering, Imperial College London, South Kensington Campus, LondonSW7 2AZ, U.K.

## Abstract

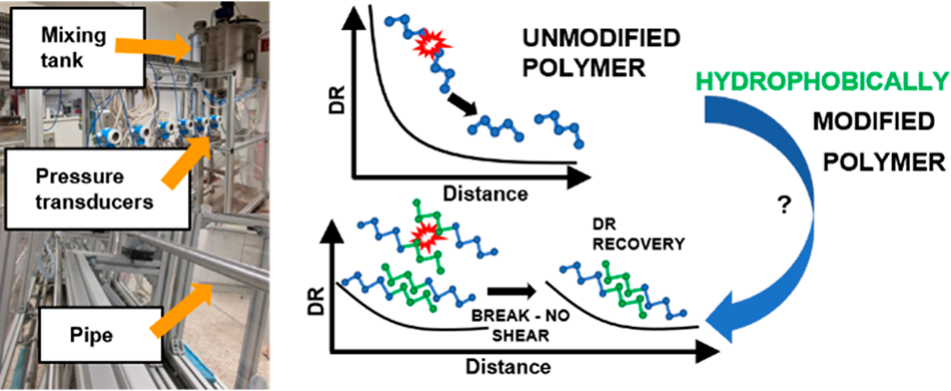

Hydrophobically modified associating polymers could be effective drag-reducing agents containing weak “links” which after degradation can reform, protecting the polymer backbone from fast scission. Previous studies using hydrophobically modified polymers in drag reduction applications used polymers with *M*_*w*_ ≥ 1000 kg/mol. Homopolymers of this high *M*_*w*_ already show significant drag reduction (DR), and the contribution of macromolecular associations on DR remained unclear. We synthesized associating poly(acrylamide-*co*-styrene) copolymers with *M*_*w*_ ≤ 1000 kg/mol and various hydrophobic moiety content. Their DR effectiveness in turbulent flow was studied using a pilot-scale pipe flow facility and a rotating “disc” apparatus. We show that hydrophobically modified copolymers with *M*_*w*_ ≈ 1000 kg/mol increase DR in pipe flow by a factor of ∼2 compared to the unmodified polyacrylamide of similar *M*_*w*_ albeit at low DR level. Moreover, we discuss challenges encountered when using hydrophobically modified polymers synthesized via micellar polymerization.

## Introduction

Fully developed turbulence in pipe flows produces frictional drag resulting in dissipation of the input energy driving the flow. Fluid friction can be significantly reduced by the addition of minute amounts of polymers (10–100 wppm) causing an increase in flow rate for a given pressure gradient.^1^ This phenomenon, referred to as polymer drag reduction (DR), implies that the pressure drop for dilute polymer solutions passing through a pipe will be notably lower than for the pure solvent at the same flow rate. For this reason, polymer-induced drag reduction is of immense interest for industrial applications, such as efficient pipeline transport of fluids,^2^ hydraulic fracturing and drilling operations,^3,4^ sewers,^5^ fire-fighting,^6^ irrigation systems,^7^ and drift control during spraying in agriculture.^8^

Virk^9^ showed that the molecular weight of flexible polymers is the essential property determining the effectiveness of a polymer drag-reducing agent. It was shown that the shear wall stress in pipe flow is inversely proportional to the molecular weight of the polymer drag-reducing agent.^9,10^ Besides molecular weight, other parameters such as polymer chain flexibility, concentration, molecular structure, and polymer conformation in solution affect drag reduction.^11−14^ For water poly(ethylene oxide) (PEO) and polyacrylamide (PAAm) are most effective, and PAAm is the most commercially used polymeric drag reducer.^9,15−20^ Unfortunately, drag-reducing polymers lose effectiveness due to mechanical degradation caused by high shear in turbulent flow.^21^ The mechanism of drag reduction is closely related to polymer degradation.^22,23^ Horn and Merrill^24^ studied the degradation behavior of linear polymers and unveiled that high-molecular-weight polymers are more sensitive to degradation compared with lower molecular weight ones. They observed that macromolecules are greatly stretched before they break, causing backbone scission near the chain midpoint, producing a narrower molecular weight distribution. Similar findings were reported by others.^25−27^ But broader distributions of breaking points are also possible.^28^ On the other hand, aggregates might be more effective drag reducers than individual polymer molecules since they form structures of higher molecular weight.^4^ Cox et al.^29^ reported that aggregates of molecules may be important for polymer DR effectiveness since shear is expected to break aggregates rather than the polymer backbone. Aggregations can reform and therefore promote long-term DR. Such polymer associations do occur in aqueous solutions of hydrophobically modified water-soluble polymers, which form micellar structures^30−36^ or a “*temporary three-dimensional network in aqueous solutions*”,^30^ which affect the viscosity at various shear rates, offering the potential to be useful drag-reducing agents in fully developed turbulent pipe flow.^37−41^ We wish to test the hypothesis that high-molecular-weight associations formed by lower molecular weight hydrophobically modified water-soluble polymers can act as efficient drag-reducing agents. Thus far, studies on drag reduction using associating polymers focused only on polymers with molecular weights *M*_*w*_ ≥ 1000 kg/mol, showing them to be good drag-reducing agents. But already McCormick et al.^41^ pointed to the necessity to test associating polymers with lower individual molecular weights at which nonassociating homopolymers do not display drag reduction. This will allow to gain a better understanding of the effect of hydrophobic high-molecular-weight associations on drag reduction.

Investigations of turbulent drag reduction are commonly performed either using various rotating “disc” apparatus^25,42−45^ or in pipe flow setups.^2,24^ However, the flow pattern in both experimental setups is different. To gain comprehensive insights into polymer-induced drag reduction, an appropriate combination of measuring techniques is of utmost importance. We set out to test the influence of the association behavior of hydrophobically modified polymers on drag reduction performance at application-relevant conditions using a pilot-scale flow facility. We use a pressure-driven horizontal pipe flow device to simulate long pipelines and thus ensure real-time drag reduction measurements. In addition, we use a double-gap geometry to compare polymer DR behavior in a rotating “disc” apparatus to that in a horizontal pipe. We synthesized poly(acrylamide-*co*-styrene) with molecular weights at which pure PAAm does not produce significant drag reduction and varied the hydrophobe content in the polymer, since associative properties are more pronounced with increasing content of hydrophobic moieties^30^ and incorporation of aromatic groups.^40^ We present the effect of hydrophobe content in the polymer main chain on the association, rheological, and drag reduction behavior.

## Experimental Section

### Materials

Acrylamide (AAm) (≥99%, Sigma-Aldrich) was recrystallized twice from acetone (≥99.5%, Donauchem). Styrene (St) (≥99%), potassium persulfate (K_2_S_2_O_8_, ≥99%), hexadecyltrimethylammonium bromide (CTAB, ≥98%), sodium azide (NaN_3_, ≥99.5%), sodium nitrate (NaNO_3_, ≥98%), deuterium oxide (D_2_O, 99.9 atom % D), formamide (≥99%), and magnesium sulfate (MgSO_4_, ≥99.5%) were purchased from Sigma-Aldrich and used without further purification. Methanol (≥99.8%, Fisher Scientific) and potassium chloride (KCl, 99.5%, BDH, VWR) were used as received. Polyacrylamides with nominal molecular weights *M*_*w*_ = 0.5 (PAAm0.5) and *M*_*w*_ = 1.0 × 10^6^ g/mol (PAAm1) were kindly provided by SNF Floerger (Andrézieux, France). Poly(ethylene oxide) (*M*_*w*_ = 24 × 10^3^ g/mol, PEO-24K) and dextran (*M*_*w*_ = 69 × 10^3^ g/mol, Dextran-T69K) standards were purchased from Malvern. Dishwasher salt (sodium chloride, NaCl) (Tandil) was bought from Hofer. Nitrogen (≥99.999%, Messer) was used to provide an inert atmosphere during synthesis. The dialysis tubing (Bio Design, Thermo Fisher Scientific) used for purification of polymers had a molecular weight cutoff (MWCO) of 8000 Da. Deionized water (conductivity of 0.055 μS/cm, Water Purifier, Elga, UK) was used for all experiments, except for the flow facility.

### Synthesis of Poly(acrylamide-*co*-styrene)

Poly(acrylamide-*co*-styrene), P(AAm-*co*-St), copolymers were synthesized via micellar polymerization adapting a previously reported method^36^ to vary the hydrophobe content in the polymer chain. PAAm, without hydrophobe incorporated, was also synthesized as a reference for the structure analysis using NMR. Either 3.50 g (9.60 mmol) or 1.48 g (4.06 mmol) of CTAB was dissolved in 50 mL of deionized water in a round-bottom flask under magnetic stirring followed by addition of 37.27 mg (0.36 mmol) or 74.54 mg (0.72 mmol) of styrene for 1 mol % or 2 mol % hydrophobe content in the copolymer. The mixture was stirred overnight to enable micellization. Afterward, 2.50 g (35.17 mmol) of AAm was dissolved in the reaction mixture for ∼30 min. The mixture was degassed by three freeze–pump–thaw cycles using a Schlenk line. The initiator solution was prepared by dissolving K_2_S_2_O_8_ (88.25 mg, 0.33 mmol) in 2 mL of deionized water. This solution was degassed by nitrogen injection. The reaction mixture was heated to 80 °C, and the initiator solution injected using a degassed syringe. This mixture was continuously purged with nitrogen. After 24 h the reaction was stopped by cooling in ice water. The polymer was precipitated by dropwise addition into methanol under gentle stirring. The white precipitate was stirred in methanol overnight, and the solid removed by filtration, redissolved in deionized water, and again precipitated in methanol. This purification process was repeated twice. To purify the polymer further, it was dissolved in water and then dialyzed against ∼4 L of deionized water for 10 d. The polymer was recovered by freeze-drying.

The polymerization yield (*Y*) was determined as follows:
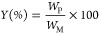
1where *W*_P_ is the weight (g) of the purified polymer and *W*_M_ that of the monomers. The yields for all the synthesized polymers after dialysis ranged between 85% and 95%.

The copolymers are denoted as 1P(AAm-*co*-1St), 0.7P(AAm-*co*-1St), and 0.5P(AAm-*co*-2St); the first number denotes their molecular weight in 10^6^ g/mol as determined by gel permeation chromatography (see Table 1) and the second the molar ratio of St/AAm, which was either 1 or 2 mol %.

**Table 1:** Weight Averaged (*M*_*w*_) and Viscosity Averaged (*M*_η_) Molecular Weight, Polydispersity Index *Đ*, Molecular Dimensions in Solution, Namely, Radius of Gyration *R*_g_, Hydrodynamic Radius *R*_H_, Viscosity Averaged Hydrodynamic Radius *R*_η_, Polymer Volume Fraction in Solution φ, and 2(Osmotic) Viral Coefficient *A*_2_^a^

sample	*M*_η_(kg/mol)^b^	*M*_*w*_(kg/mol)^c^	*Đ*	*M*_*w*_(kg/mol)^d^	*R*_*g*_ (nm)	*A*_2_ × 10^–7^(mol dm^3^/g^2^)	*R*_η_ (nm)	*R*_H_ (nm)	φ (%)^e^
PAAm1	1030^f^	1134 ± 54	2.0 ± 0.1	1000 ± 50^f^	70 ± 3^f^	5.9 ± 0.1^f^	37^f^	31 ± 1^f^	1.2^f^
	950^g^			1400 ± 60^g^	86 ± 2^g^	3.0 ± 0.7^g^	35^g^	68 ± 2^g^	1.2^g^
1P(AAm-*co*-1St)	1310^f^	1022 ± 30	1.4 ± 0.0	1180 ± 70^f^	84 ± 4^f^	3.9 ± 0.8^f^	42^f^	24 ± 1^f^	1.5^f^
	1370^g^			3200 ± 290^g^	136 ± 4^g^	4.3 ± 0.4^g^	43^g^	118 ± 1^g^	1.5^g^
PAAm0.5	310^f^	290 ± 3	2.1 ± 0.0	200 ± 10^f^	27 ± 7^f^	6.8 ± 0.4^f^	18^f^	16 ± 1^f^	0.5^f^
	190^g^			880 ± 160^g^	110 ± 8^g^	5.3 ± 1.3^g^	14^g^	37 ± 1^g^	0.3^g^
0.7P(AAm-*co*-1St)	720^f^	730 ± 40	1.5 ± 0.1	970 ± 70^f^	113 ± 5^f^	1.7 ± 0.3^f^	30^f^	28 ± 1^f^	0.9^f^
	470^g^			2360 ± 680^g^	148 ± 13^g^	4.9 ± 0.8^g^	23^g^	110 ± 1^g^	0.7^g^
0.5P(AAm-*co*-2St)	500^f^	510 ± 30	1.4 ± 0.0	600 ± 10^f^	54 ± 2^f^	3.4 ± 0.2^f^	24^f^	20 ± 2^f^	0.7^f^
	510^g^			20600 ± 4700^g^	224 ± 13^g^	2.5 ± 0.2^g^	24^g^	131 ± 1^g^	0.7^g^

a The quantities were extracted from static and dynamic light scattering, gel permeation chromatography, and rheology.

b–d Weight-averaged molecular weight obtained using rheometer, gel permeation chromatography, and static light scattering, respectively.

e Calculated for a polymer concentration of 0.01 wt %.

f Data obtained in formamide.

g Data obtained in aqueous 0.025 M MgSO_4_.

### Characterization of Poly(acrylamide-*co*-styrene)

^1^H NMR spectra were acquired in D_2_O using a 600 MHz NMR (Bruker BioSpin, Rheinstetten, Germany) and processed using TopSpin 4.0.9. The amount of incorporated styrene in P(AAm-*co*-St) was calculated as follows:
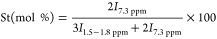
2where *I*_7.3 ppm_ represents an integral of a triplet corresponding to protons of phenyl groups (−C_6_H_5_) and *I*_1.5–1.8 ppm_ an integral of protons corresponding to methylene (−CH_2_−) groups present in the polymer backbone.

### Dynamic and Static Light Scattering

Dynamic (DLS) and static light scattering (SLS) were performed using a compact goniometer system (ALV/CGS-3, Langen, Germany) equipped with a 22 mW helium–neon laser (632.8 nm). Samples for light scattering measurements were prepared as follows: the polymer was dissolved in formamide to obtain nonassociated polymer solutions as well as in an aqueous solution of 0.025 M MgSO_4_ to allow for polymer associations to form by screening any residual charges. Stock solutions were first shaken at 200 rpm using an orbital shaker (PSU-10i, Biosan) for 48 h. Then they were heated for an additional 48 h at 50 °C accompanied by mechanical mixing at 100 rpm using a magnetic stirrer (RCT basic, IKA) equipped with a temperature sensor (PT 1000.60). The stock solutions were diluted to concentrations ranging from 0.005 to 0.06 wt % for those prepared in aqueous MgSO_4_ solutions, while those prepared in formamide from 0.01 to 0.2 wt %. Prior to DLS and SLS measurements, all the solutions were filtered through 0.45 μm filters (CHROMAFIL Xtra PVDF-45/25) to remove dust particles present. To eliminate any impurities, the filters were always first rinsed with solvent followed by flushing the filters with ∼2 mL of a sample solution to prevent polymer retention by the filters.

The molecular weights of single polymer molecules and macromolecular associations were determined via SLS. The SLS measurements were carried out at scattering angles (30–130°) in 10 angular steps. The refractive index increment (d*n*/d*c*) was determined using a differential refractometer (Brookhaven BI-DNDC). A minimum of five concentrations was used to determine the apparent weight-average molecular weight *M*_*w*_, radius of gyration *R*_g_, and second virial coefficient *A*_2_ using either the Zimm or Berry data reduction method, processed by ALV-Fit and Plot software. Data points at low angles that were considerably deviating from linearity were excluded in the analysis.

DLS measurements were performed at a goniometer angle of 90° to determine the hydrodynamic radii (*R*_H_) of polymer molecules/aggregates. The measured correlation function was transformed into the translational diffusion coefficient *D* and converted into *R*_H_ using the Stokes–Einstein relation:
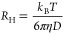
3where *k*_B_ is the Boltzmann constant, *T* the absolute temperature, and η the viscosity of the solvent.

### Rheological Characterization

The rheological measurements were carried out using a rheometer (TA Instruments Discovery HR-2) at 25 ± 0.1 °C. The temperature was controlled using a water circulatory thermostat (Thermo Cube). Linear viscoelasticity and dynamic experiments were performed. The rheometer was also used as a rotary “disk” apparatus to assess DR capabilities of polymer solutions on a small scale. Stock solutions were prepared at concentrations of approximately 8 wt % polymer in water. MgSO_4_ was added to the dilute solutions to a concentration of 0.025 M. Experiments with polymer concentrations ranging from 0.0001 to 0.9 wt % were performed in a double-gap cylindrical geometry (*d*_1_ = 30.20 mm, *d*_2_ = 32.03 mm, *d*_3_ = 34.99 mm, *d*_4_ = 37.00 mm, and rotor height *L* = 55.00 mm), while for polymer solutions with concentrations from 2 to 8 wt % a cone–plate geometry (*d* = 40.00 mm, cone angle 1°) was used. Sample volume in the double-gap geometry was 12 mL, and 0.3 mL in cone–plate geometry. Viscosity measurements were performed at a constant shear rate of 10 s^–1^ for 90 s. The runs were repeated four times and values averaged. The shear rate dependence of viscosity was acquired in a shear rate-controlled mode. The shear rate was increased in steps, and when equilibrium was reached, data were taken for 10 s. Three shear rate intervals were chosen for these experiments depending on solution viscosity (concentration): 1–120 s^–1^, 1–140 s^–1^, or 1–1000 s^–1^. The limit of the linear viscoelastic region was determined prior to the dynamic measurements using a strain-controlled amplitude sweep. The dynamic measurements were conducted at a frequency of 1–200 rad/s^–1^. All measurements were performed at a constant strain of 0.1 that led to a linear response.

The viscosity averaged molecular weight *M*_η_ was determined from the measured viscosities at dilute concentrations using the Mark–Houwink equation. We used the Mark–Houwink parameters *K* = 9.33 × 10^*–*3^ cm^3^/g and α = 0.75 determined in 0.1 M NaCl solution,^46^ as its ionic strength is equivalent to that of 0.025 M MgSO_4_. The intrinsic viscosity [η] was determined from measured viscosities as follows:^47^
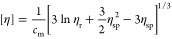
4where *c*_m_ is the polymer mass concentration in g/mL and η_r_ the relative and η_sp_ the specific viscosity. The viscosity averaged hydrodynamic radii *R*_η_ were calculated from [η]
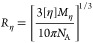
5where *N*_A_ is Avogadro’s number.

Errors for *M*_η_, *R*_η_, and φ calculated from viscosities data were lower than 0.001% and thus are not presented in Table 1.

DR characterization in Taylor flow on a small characteristic length scale was performed in the rheometer equipped with the double-gap geometry using the method described by Nakken et al.^44^ and Pereira et al.^18^ The double-gap measuring cell has a large contact area and, therefore, allows for good reproducibility.^18^ The onset of Taylor flow is visible by an abrupt change of the slope of the function η = *f*(γ̇) where γ̇ is the shear rate. Taylor flow onset was detected by increasing the shear rate in steps from 100 to 3000 s^–1^, enabling equilibrium at each step. The critical shear rate γ̇_c_ at Taylor flow onset was determined by a piecewise linear fit to η = *f*(γ̇) in the region around γ̇_c_. γ̇_c_ was then converted to the critical angular velocity ω_c_:^18^
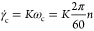
6where *n* is the speed of rotation and *K* a geometrical factor: , where *R*_*i*_ are the radii of the geometry. The apparent viscosity at the Taylor flow onset was calculated as follows:^44^
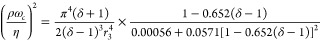
7where ρ is the solution density, η the apparent viscosity at onset, and δ the ratio of the distance between rotor and stator δ = *R*_4_/*R*_3_ = *R*_2_/*R*_1_.

DR is defined as
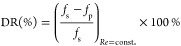
8where *f*_s_ and *f*_p_ are the Fanning friction factors of pure solvent and polymer solution. *f* is calculated as follows:^18^
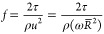
9where τ is the shear stress, *u* the linear velocity, and *R̅* the mean radius: .

τ was determined from the measured torque exerted on the rotor *T*:
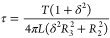
10

The Reynolds number *Re* for the flow in the double-gap geometry is defined as^18^
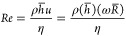
11where *ωR̅* is the characteristic velocity and *h̅* the average gap width calculated as follows: .

### Vienna Experiment for Drag-Reducing Agents

The characterization of the DR capability in turbulent pipe flow was performed in our pilot-scale flow facility called Vienna Experiment for Drag Reducing Agents (ViEDRA). Large volumes (up to 300 L) of polymer solutions were prepared in a mixing tank. Compressed air was used to drive the solution through a system of pipes and hoses of a total length of ∼20 m per cycle. This includes a 7.2 m long stainless-steel pipe with an inner diameter of 26 mm representing the test section. Along this test section six differential pressure sensors are located (Deltabar S, Endress+Hauser, Germany). The measured pressures allow for calculation of the fanning friction factor *f* using the Darcy–Weisbach relation:
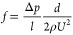
12where Δ*p* is the pressure drop along the test section, *l* the length of the test section, *d* the pipe diameter, ρ the solution density, and *U* the volume averaged velocity.

The flow rate is measured using a magneto-inductive flowmeter (Sitrans F M Magflo MAG5000, Siemens, Denmark). A feedback controller adjusts the driving pressure and allows for experiments at constant flow rate and thus with constant .

Each polymer solution was cycled multiple times through ViEDRA in order to investigate the degradation of the DR agents. Details on the flow device will be published elsewhere.

### Gel Permeation Chromatography

*M*_*w*_ and polydispersity *Đ* of the polymers were determined using a triple detection GPC system (Viscotec TDA 302, Malvern Panalytic) equipped with a solvent reservoir, a degasser (CSI6150 4 channel degasser, laserchrom), a high-performance liquid chromatography (HPLC) pump (LC-20 ADVP, Shimadzu Deutschland GmbH), and an automatic sample injector (S5200 Sykam GmbH). The polymer molecules were separated according to their hydrodynamic volumes using a guard column (A-Guard, Viscotek, Malvern) and two analytical columns (A4000 with an exclusion limit of 1 × 10^6^ g/mol and A6000 M with an exclusion limit of 20 × 10^6^ g/mol). The postcolumn filter had a pore size of 0.2 μm (Nylon Membrane Filters, Whatman). A PEO standard (*M*_*w*_ = 23 651 g/mol) in combination with a validation standard of dextran (*M*_*w*_ = 68 991 g/mol) was used to calibrate the detectors. The temperature was kept constant at 30 °C. The aqueous eluent contained 0.1 mol/L NaNO_3_ and 0.02 wt % NaN_3_. The flow rate during the measurements was kept constant at 0.7 mL/min. Prior to the measurements, the polymer samples were filtered through a 0.45 μm filter. The chromatograms were analyzed using the OmniSEC 5.02 software.

## Results and Discussion

**Figure 1:**
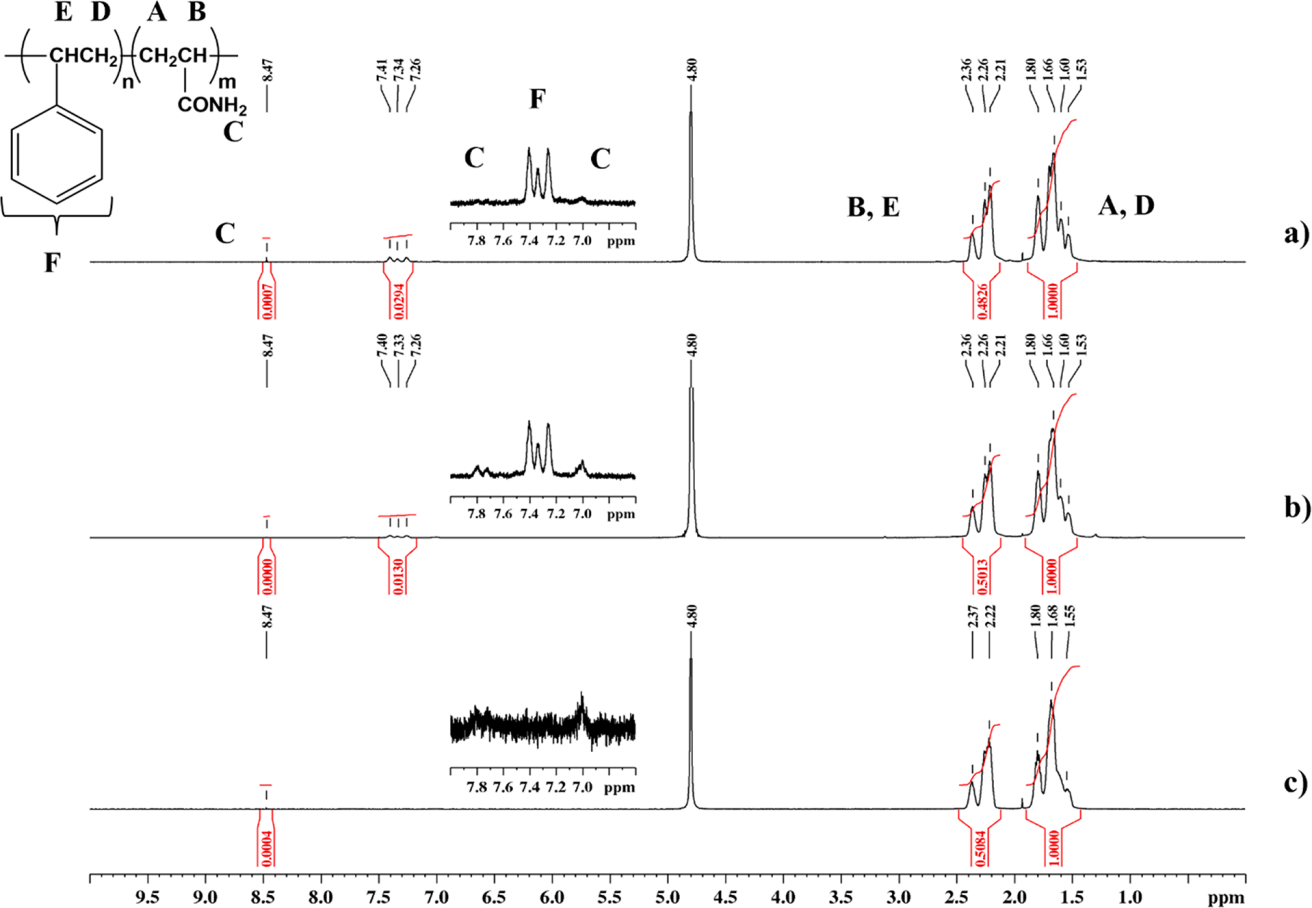
^1^H NMR spectra recorded in D_2_O for (a) 0.5P(AAm-*co*-2St), (b) 0.7P(AAm-*co*-1St), and (c) PAAm homopolymer.

^1^H NMR spectra (Figure 1) of hydrophobically modified and pure PAAm exhibited strong signals at ∼1.53–1.80 and 2.21–2.37 ppm corresponding to methylene (−CH_2_−) and methine (>CH−) protons present in the polymer backbone, while the weak signals at ∼7.0, 7.8, and 8.47 ppm were assigned to amide groups (−CONH_2_). The signal at 4.80 ppm corresponds to solvent D_2_O (or HOD). Copolymers additionally exhibited a weak triplet in the range 7.26–7.41 ppm corresponding to the protons of phenyl groups (−C_6_H_5_), which is stronger for 0.5P(AAm-*co*-2St), which contained ∼2 (actual amount 1.92) mol % St (Figure 1a) in its backbone when compared to 0.7P(AAm-*co*-1St), containing 0.86 mol % St (Figure 1b). It is noteworthy that the amounts of St incorporated in P(AAm-*co*-St) were in good agreement with the amounts of St added to the reaction mixture. The ^1^H NMR spectra of 0.7P(AAm-*co*-1St) and 1P(AAm-*co*-1St) are identical (see SI, Figure S1), and the St content in 1P(AAm-*co*-1St) was found to be 0.92 mol %. Elemental analysis confirmed that the polymers comprised C, H, N, and O (Table S1). Additionally, bromine (Br) from CTAB was detected, which remained in the sample after purification. The Br amount was found to be <0.01 wt %, indicating that most CTAB was successfully removed during polymer purification.

### Molecular Dimensions

The molecular weight, molecular dimensions in solution, and second virial coefficient *A*_2_ are summarized in Table 1. Using SLS we determined *M*_*w*_, *R*_g_, and *A*_2_. *M*_*w*_ was also obtained using GPC. In addition, we calculated *M*_η_ and *R*_η_ from measured viscosities. Two commercial PAAm with nominal molecular weights 500 and 1000 kg/mol, similar to those synthesized, were utilized as control samples; their *M*_*w*_ obtained using GPC were 290 ± 3 and 1134 ± 54 kg/mol, respectively. Macromolecular associations, which formed in 0.025 M MgSO_4_ of 1P(AAm-*co*-1St) and 0.7P(AAm-*co*-1St), had *M*_*w*_’s of 3200 ± 290 and 2360 ± 680 kg/mol, respectively, which is approximately two or three times higher than the *M*_*w*_ of individual molecules as determined by GPC and SLS in formamide. 0.5P(AAm-*co*-2St), having a *M*_*w*_ of single chains of 600 ± 10 kg/mol in formamide (Figure S2), formed macromolecular associations in aqueous MgSO_4_ solution (Figure S3) with a *M*_*w*_ of 20 600 ± 4700 kg/mol. It is worth noting that the polymer molecular weights determined by different measurements were in quite good agreement.

The hydrophobically modified polymers synthesized as well as the commercial control PAAm can be classified into two groups with similar molecular weights (*M*_*w*_ ≈ 1000 kg/mol and *M*_*w*_ < 1000 kg/mol). It is important to point out that these two groups were selected on the basis of *M*_*w*_ determined by GPC measurements. The reason for selecting GPC *M*_*w*_ data is that we tracked *M*_*w*_ of polymers during DR measurements in ViEDRA using GPC. In all cases the values of *A*_2_ were positive. Thus, all polymers were dissolved in “good” solvents in the dilute range having a polymer volume fraction in solution φ < 0.20^48^ for subsequent drag reduction measurements. From SLS it is obvious that all polymers tend to associate in aqueous 0.025 M MgSO_4_ solution (see Table 1). This is more pronounced for polymers containing hydrophobic blocks and for shorter individual chains. *R*_H_ from DLS (Figure S4) support these observations. The molecular weight derived from viscosity *M*_η_ is not affected when changing from formamide to aqueous MgSO_4_. *R*_η_ remains approximately constant. Possibly the shear rate of γ̇ ≤ 200 s^–1^ is already sufficiently high to break up hydrophobic associations.

### Rheological Properties

The viscoelastic behavior of the polymers was characterized by measuring the concentration dependence of zero-shear viscosity η_0_, the shear rate dependence of apparent viscosity η, and frequency dependencies of the storage *G*′ and loss *G*″ moduli. The polymer dynamics is greatly influenced by configurational interactions that restrict movement of polymer chains.^49^ These configurational restrictions are not prominent in dilute solutions, but the dynamic polymer properties are controlled by translation and rotation of the chains.^49^ Due to low polymer *c* in dilute solutions, their viscosity is governed by the solvent viscosity and only the weak polymer contribution depends on *c*. At high polymer *c* for which η_0_ ≫ η_solvent_, the η_0_ increases steeply as described by the power law η_0_ ∼ *c*^α^ with α = 3.75.^49^ This steep increase in viscosity is caused by polymer molecular entanglements^49^ affecting the polymer diffusion. We can distinguish dilute and semidilute entangled regimes based on η_0_ = *f*(*c*).^33^ The transition between these two concentration regimes is defined as a semidilute unentangled regime.^33^ The semidilute unentangled regime sets in at the first abrupt change in slope of η_0_ = *f*(*c*) denoting the overlap of polymer chains, with c* being the overlap concentration. The second change in slope represents entanglement of polymer chains with the corresponding concentration being the entanglement concentration *c*_e_, and the semidilute entangled regime comes into play. Viscosity of polymer solutions in the entangled state will depend on polymer chain length referring to the molecular weight via the relation η_0_ ∼ *M*^α^ with α = 3.^49^

**Figure 2:**
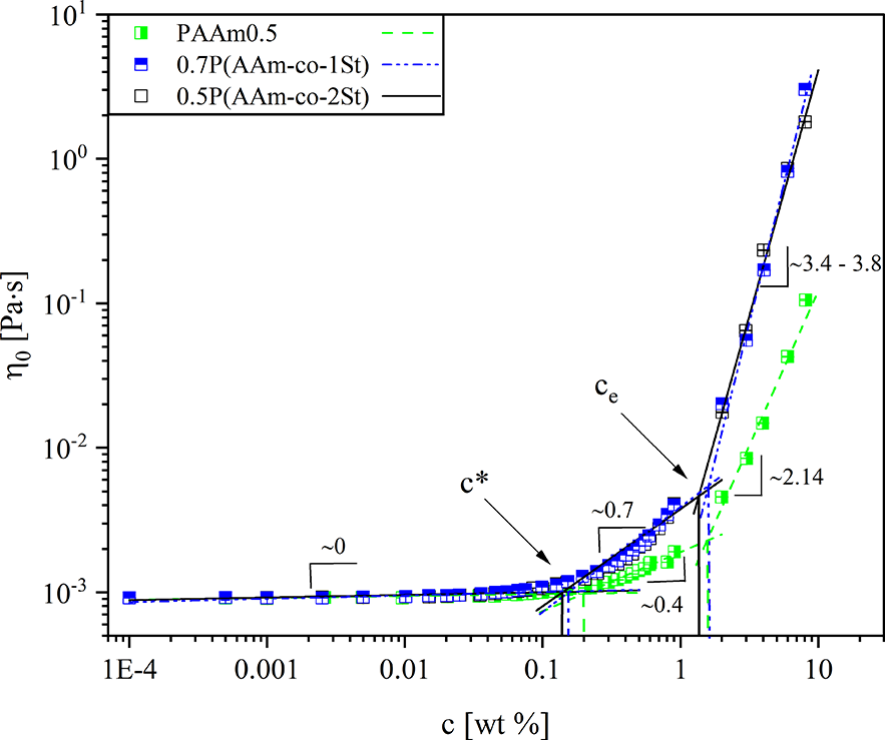
Influence of the hydrophobe amount on concentration *c* dependence of the zero-shear viscosity η_0_ determined in aqueous 0.025 M MgSO_4_ for polymers with *M*_*w*_ < 1000 kg/mol.

Regalado et al.^33^ related c* of unmodified polymers to the concentration where hydrophobically modified copolymers start to aggregate. Thus, we compared the concentration dependence of η_0_ of 0.7P(AAm-*co*-1St) and 0.5P(AAm-*co*-2St) to that of PAAm0.5 (Figure 2). The two copolymers having different hydrophobe amounts exhibited similar c* obeying the relation c* ∝ *M*^–0.8^, which agrees with the observations made by Regalado et al.^33^c* was scaling with molecular weight of polymer molecules, and for PAAm0.5 c* shifted to higher values compared to the copolymers. For all polymers *c*_e_ was found to be similar and the ratio of *c*_e_/c* was in the range of 5–10, which agrees with the literature.^33^ At *c* > *c*_e_ the copolymers followed the power law (η_0_ ∼ *c*^3.75^) displaying slopes close to theoretical values (Table S2). PAAm0.5 had a slope of 2.14 ± 0.19, indicating that polymer chains are too short for entanglements to govern its viscoelastic behavior. This observation agrees with the exponent range^50^ established for systems consisting of short chains that are marginally affected by entanglements 2.5 ≥ α ≥ 1.

In experiments on shear rate dependence of η in the semidilute entangled concentration regime (Figure S6) we observed shear thinning for 0.7P(AAm-*co*-1 St) and 0.5P(AAm-*co*-2St) at *c* ≥ 4 wt %, while no shear thinning was observed for PAAm0.5 in the tested concentration range. η of 0.7P(AAm-*co*-1St) and 0.5P(AAm-*co*-2St) in the semidilute entangled region were in approximately the same range, except for *c* = 8 wt %, where 0.7P(AAm-*co*-1St) had a η about ∼2 higher than 0.5P(AAm-*co*-2St). Our observations are broadly in line with those reported by Martínez Narváez et al.^51^ for hydrophobically modified hydroxyethyl cellulose. *G*′ and *G*″ (Figure S7) for 0.5P(AAm-*co*-2St) were approximately in the same range as for 0.7P(AAm-*co*-1St). The frequency dependences of *G*′ and *G*″ (Figure S7) for PAAm0.5 indicated that the crossover point of *G*′ and *G*″—different from 0.7P(AAm-*co*-1St) and 0.5P(AAm-*co*-2St)—was not reached in the examined *c* and ω range. *G*′ and *G*″ are one magnitude of order lower than for other polymers tested. Overall, the dynamic properties of the investigated polymers scaled with *M*_*w*_ of the polymers.

PAAm1 already followed the power law ln η = α ln *c* (Table S2) in accordance with the behavior of the modified polymers 0.7P(AAm-*co*-1St) and 0.5P(AAm-*co*-2St) (Figures S6a and S7a). Therefore, we did not perform the rheological analysis of the 1P(AAm-*co*-1St) in the semidilute entangled regime (Figure S5).

### Drag Reduction Behavior

**Figure 3:**
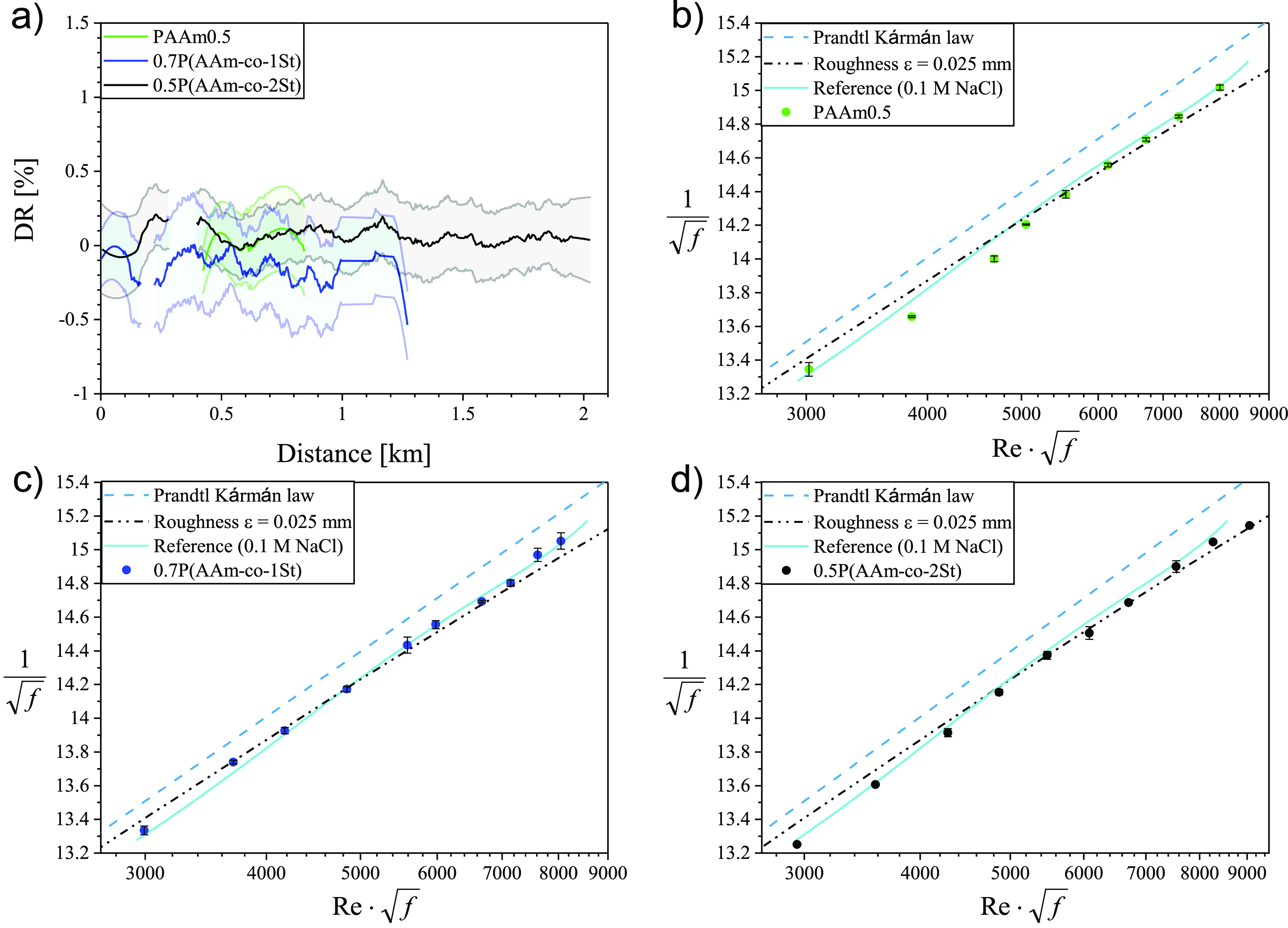
(a) Drag reduction DR(%) performance of polymers with *M*_*w*_ < 1000 kg/mol as a function of traveled distance through ViEDRA at constant polymer concentration *c* = 0.01 wt % and *Re* = 100 000. Prandtl–Kármán plots for (b) PAAm0.5, (c) 0.7P(AAm-*co*-1St), and (d) 0.5P(AAm-*co*-2St). The dash double dot line represents the influence of pipe surface roughness.

**Figure 4:**
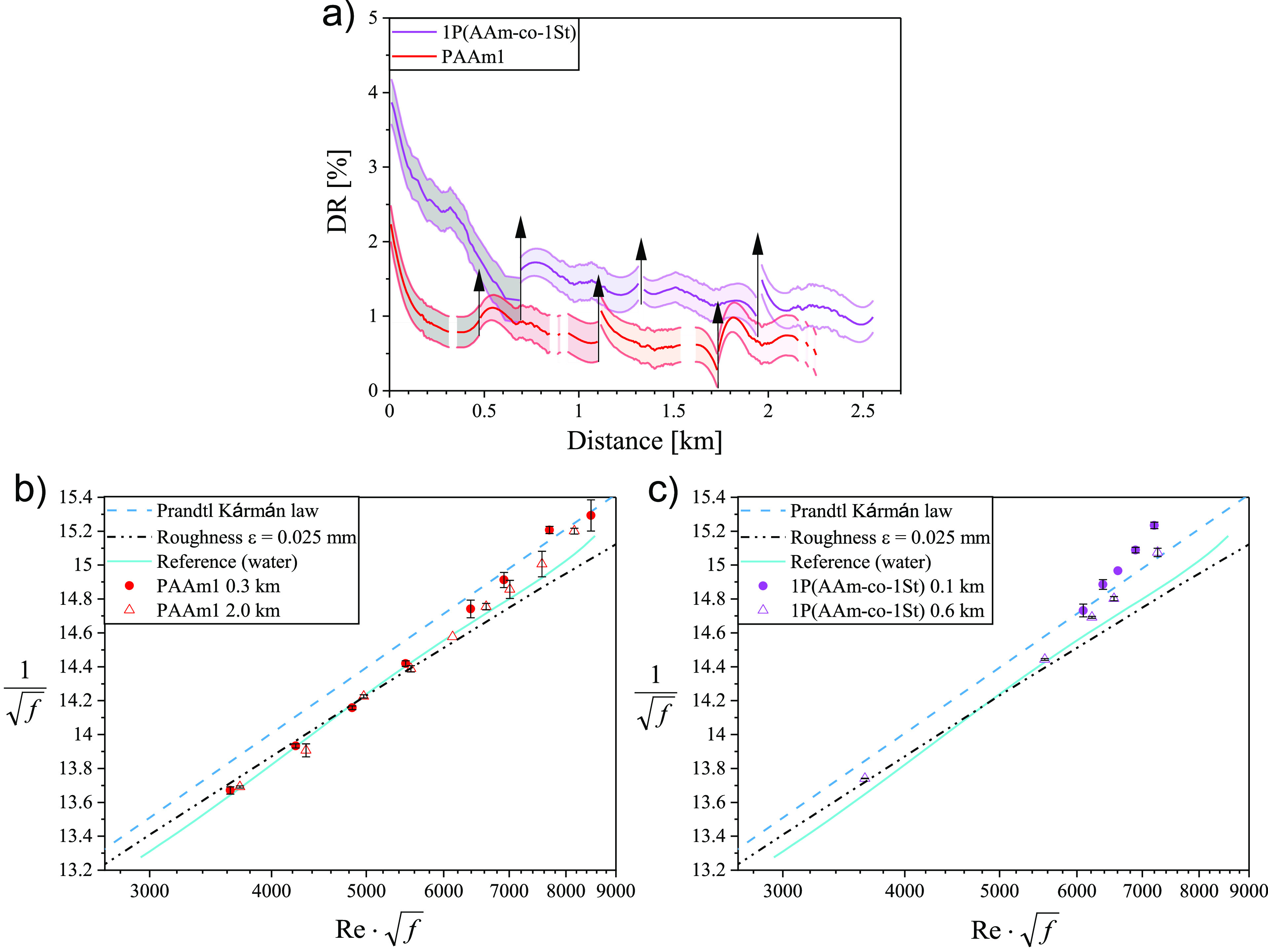
(a) Drag reduction DR(%) of polymers *M*_*w*_ ≈ 1000 kg/mol as a function of distance traveled through the test section at constant polymer *c* = 0.01 wt % and *Re* = 100 000. The arrows pointing upward represent points of salt addition. Prandtl–Kármán plots for (b) PAAm1 after 0.3 and 2.0 km represented by closed and open symbols and (c) 1P(AAm-*co*-1St) 0.1 and 0.6 km illustrated by closed and open symbols.

We assessed the DR performance of the hydrophobically modified PAAm in pipe flow and compared it with commercial PAAm of similar molecular weight. We performed these experiments at a constant polymer concentration of 0.01 wt % and constant *Re* = 100 000. *c* = 0.01 wt % was chosen for two reasons: first, the SLS analysis showed the formation of associations in the *c* range between 0.005 and 0.06 wt %, and second the concentration of polymeric DR agents used in industrial applications ranges from 0.001 to 0.01 wt %.^13^ The polymer solutions were recirculated through ViEDRA to reach flow distances of up to ∼2.5 km (Figures 3a and 4a). Shown are the mean DR(%) of each cycle over travel distance (solid line) and associated variance as the shaded area in the same color. We also analyzed the dependence of DR(%) on *Re*. The results are shown as Prandtl–Kármán plots (Figures 3b–d and 4b,c). In all these graphs, the dashed lines represent the friction factor in turbulent pipe flow as described by the Prandtl–Kármán law, and the solid lines the measured flow behavior of the pure solvent. The deviation between the actual behavior of pure solvent and the Prandtl–Kármán law is assigned to pipe roughness, which is also considered and presented in Figures 3b–d and 4b,c.

DR effectiveness is known to increase with increasing polymer molecular weight.^4,52^ An increase in molecular weight results in an increased hydrodynamic volume (for simplicity ∝ *R*_g_^3^) of polymer coils in solution but is also dependent on polymer structures and solvent quality. Therefore, “ultralong” (*M*_*w*_ ≥ 1000 kg/mol) nonassociating polymers are able, already at small elongation rates, to absorb energy by stretching, causing the fluid body to oppose elongation, which results in reduced drag.^53^ The importance of elastic effects for DR was discussed by Metzner and Graham Park^54^ and Kalashnikov.^55^ Nonassociating PAAm homopolymers with *M*_*w*_ = 1400 kg/mol at *c* = 0.02 wt % exhibited a DR maximum of 5%, while considerable DR capabilities of up to 42% were achieved for polyacrylamide with *M*_*w*_ = 2500 kg/mol.^57^ McCormick et al.^41^ reported that higher *M*_*w*_ (*M*_*w*_ ≥ 1000 kg/mol) hydrophobically modified associating PAAm produce DR. Consequently, in their study the contribution of associations on DR efficiency by associating copolymers with *M*_*w*_ ≥ 1000 kg/mol was not clear.^41^ In addition, Wei et al.^53^ suggested that associating di-end-functionalized polymers, able to mimic “ultralong” polymers, must possess strongly associating functionalities at both chain ends to enable pairwise end-association. These associating polymers manifest expanded conformation in quiescent conditions that enables their elongation under flow.^53^ To test the hypothesis that high-molecular-weight associations formed by lower molecular weight hydrophobically modified water-soluble polymers act as efficient drag-reducing agents, we compared the DR efficiencies of such polymers with pure PAAm having molecular weights of *M*_*w*_ ≤ 1000 kg/mol, which do not show DR without association (Figure 3a). These experiments were performed in 0.1 M NaCl (rather than MgSO_4_ because of the amount of salt required in ViEDRA) for all three polymers. As expected, PAAm0.5 did not provide any DR(%), but also no DR(%) was seen for the hydrophobically modified PAAm of similar molecular weight independent of hydrophobe content (0.7P(AAm-*co*-1St) and 0.5P(AAm-*co*-2St)), which both did form aggregates in quiescent conditions (see Table 1). At no or very low DR(%), where *f*_s_ ≈ *f*_p_, the relative error in calculated DR(%) increased, causing fluctuations that lead to negative DR values (Figure 3a). The friction factor for pure solvent (0.1 M NaCl solution) and polymer solutions tested at various *Re*-numbers followed the Prandtl–Kármán law showing that the tested polymers possessed indeed no DR (Figure 3b–d).

The DR efficiency of PAAm1 and 1P(AAm-*co*-1St) both having *M*_*w*_ slightly above 1000 kg/mol were initially determined in water to which later NaCl was added after every ∼0.5 km to reach concentrations of 0.1, 0.5, and 1.0 M (Figure 4a) to test if salt addition does promote (re)formation of polymer associations and result in enhanced DR. In water we observed a significant decrease in DR as a function of flow distance: DR dropped from 4% to ∼1.25% for 1P(AAm-*co*-1St) and from 2.25% to ∼0.75% for pure PAAm1. We probed *M*_*w*_ of the polymers after passing through the pipe for different distances during the experiment using gel permeation chromatography (GPC) (Figure S8) and found that this decrease in DR(%) was not associated with polymer degradation. The DR(%) decrease might be due to aggregate destruction,^56^ which prevented backbone cleavage. Salt addition caused a slight increase in DR, which could indicate aggregate (re)formation for both polymers (Figure 4a). For PAAm1 this increase was followed by a fast decay, while for 1P(AAm-*co*-1St) the decay was slower.

**Figure 5:**
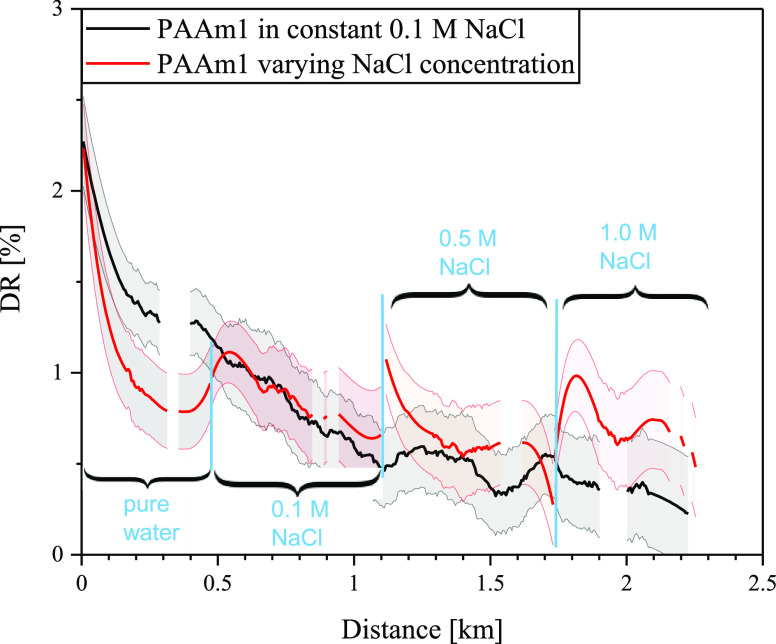
Salt influence on DR performance of reference PAAm1. Black line is for DR of PAAm1 in aqueous NaCl at constant *c* = 0.1 M and red line for DR in varying NaCl concentration.

When comparing flow distance dependence of DR(%) in the salt solution with constant salt *c* and in the salt solution with varying salt *c*, it is apparent that the salt addition had an effect already on reference PAAm promoting association formation, which resulted in higher DR(%) (Figure 5).

In addition, to test possible recovery of macromolecular associations for both PAAm1 and 1P(AAm-*co*-1St), during the experiments we allowed the polymer solutions to rest in quiescent conditions either overnight or for up to 2 days. No DR(%) increase was observed after storage at quiescent conditions. Even if the aggregates reformed during storage, it seems they are not sufficiently stable to provide improved long-term DR. Incorporating Poiseuille’s law and maximum drag reduction asymptote (MDR) established by Virk^9^ into the Prandtl–Kármán plots (Figure S9) indicates that the most pronounced DR(%) achieved by 1P(AAm-*co*-1St) was only 4%, which is in the very low DR(%) range. Hence, the use of 1P(AAm-*co*-1St) provided an improvement in initial DR, reaching 4% compared with the DR of PAAm1 (2.25%), indicating that the DR performance of the modified PAAm improved by a factor of ∼2 compared to unmodified PAAm at low DR(%). Furthermore, when plotting the *Re* dependence of DR(%) in a Prandtl–Kármán plot, a more pronounced friction reduction was observed for 1P(AAm-*co*-1St) than for PAAm1. The modified polymer shows the same DR(%) at 0.6 km as commercial PAAm at a shorter distance of 0.3 km (Figure 4b,c). It is also visible that DR(%) increases with *Re*.

The presented results indicated that hydrophobically modified polymer 1P(AAm-*co*-1St) showed higher DR capabilities at a low level compared to pure PAAm of approximately the same molecular weight (Figure 4). Even though 0.5P(AAm-*co*-2St) exhibited a high number of associations as indicated by SLS results (Table 1), its DR capabilities were in the range of reference PAAm0.5. On one hand, this result is affected by foam formation, which is more in-depth discussed below, and on the other hand, hydrophobically modified copolymers synthesized using micellar polymerization are assumed to consist of hydrophobe moieties distributed in small blocks.^58^ However, Lacík et al.^58^ argue that these copolymers suffer from compositional heterogeneity at high hydrophobe incorporation and comonomer conversion because hydrophobic monomers are being consumed and incorporated as small blocks at the early stage of the reaction, resulting in later reaction stages in homopolymer formation. This compositional heterogeneity greatly aggravates the copolymer performance as viscosity modifiers, causing no significant difference when compared with the homopolymer.^56^ Wei et al.^53^ discussed that polymers having associating functionalities distributed along their backbone form flower-like, collapsed, and rigid “supramolecules”, which do not provide the benefits of di-end-functionalized polymers for DR. The compositional heterogeneity contributes to an important aspect, which is the stability of associations in shear. Considering *R*_H_ and *R*_η_ (Table 1) it is likely that already low shear rates (γ̇ ≤ 200 s^–1^) are sufficient to destroy the aggregates. Therefore, the question is will polymers forming stronger associations in *water* (but with association strength lower than C–O bonds) produce more effective associations resulting in improved DR(%) and reduced chain scission? Wei et al.^53^ used statistical mechanics to design polymers capable of self-assembling into “megasupramolecules” with *M*_*w*_ ≥ 5000 kg/mol at *c* ≤ 0.3 wt % useful for mist control and DR in low-polarity fluids, such as liquid fuels. Theoretical estimations suggested that an optimum concentration of “megasupramolecules” of *c* > 0.005 wt % formed from long end-functionalized telechelic polymers (*M*_*w*_ = 400–1000 kg/mol) when present at concentrations of 0.14 wt %.^53^ However, the fraction of “megasupramolecules” is rather small compared to the total polymer amount. The telechelic end-functionalities provided an end-association strength between 16 and 18 *k*_B_*T*.^53^ These telechelic polymers were reported to be effective in drag reduction and mist control at *c* = 0.1 wt %, retaining their properties after 5 passes through a pump, in contrast to “ultralong” (*M*_*w*_ = 4200 kg/mol) nonassociating polymers at *c* = 0.02 wt %.^53^ Short backbone di-end-functionalized polymers (*M*_*w*_ < 400 kg/mol) resulted in small ring micelle formation and do not influence rheological properties of the solution, while very long backbones (*M*_*w*_ > 1000 kg/mol) are prone to fast degradation under strong flows.^53^ The association strength has to be ≫1 *k*_B_*T* for associations to form, but also ≪150 *k*_B_*T* (average strength of covalent bonds) to provide reversible secondary bonds acting as a “physical fuse” protecting the backbone from degradation in turbulent flows.^53^

**Figure 6:**
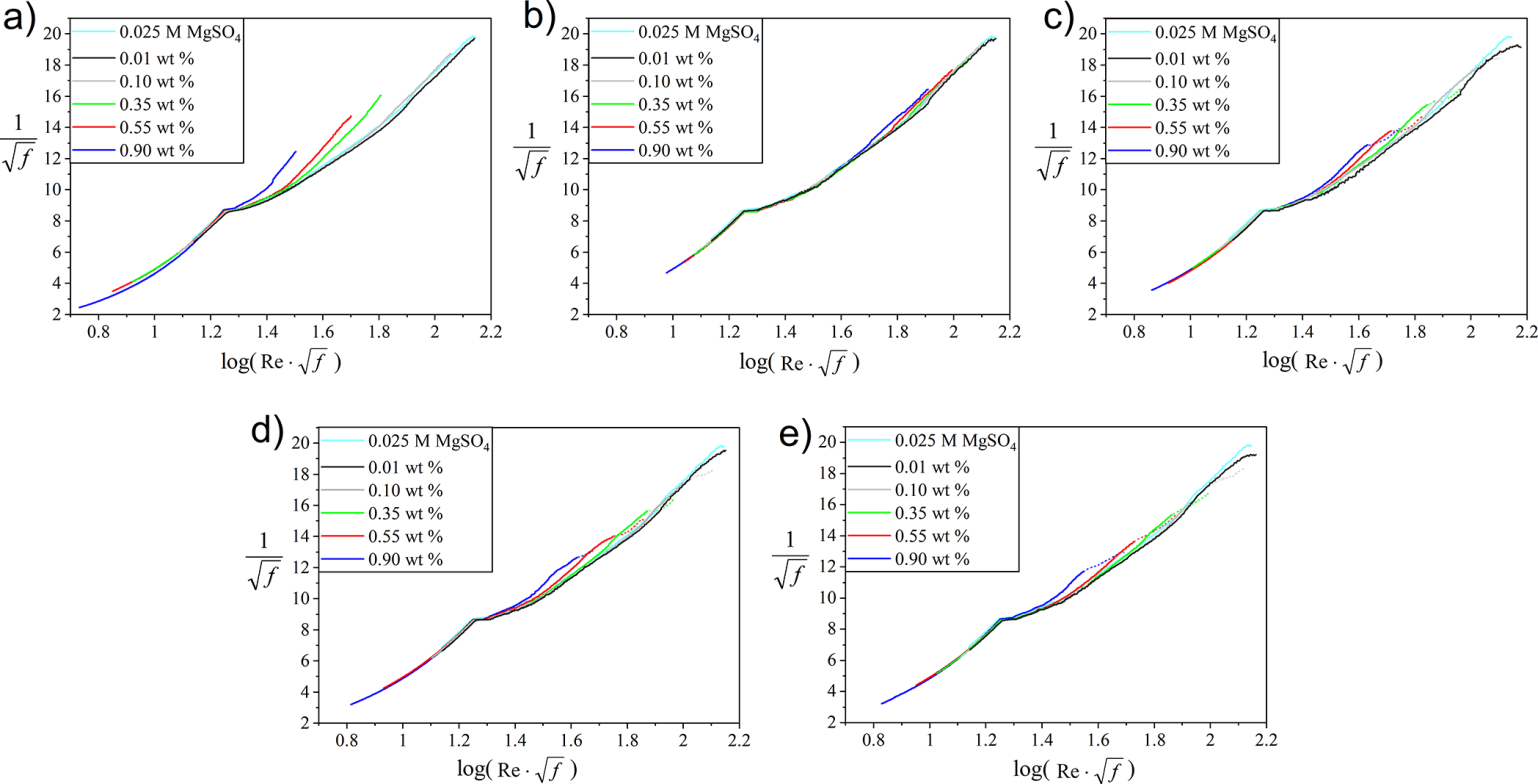
Prandtl–Kármán plots of the data obtained in a double-gap geometry at polymer *c* = 0.01, 0.1, 0.35, 0.55, and 0.90 wt % in aqueous 0.025 M MgSO_4_ for (a) PAAm1, (b) PAAm0.5, (c) 1P(AAm-*co*-1St), (d) 0.7P(AAm-*co*-1St), and (e) 0.5P(AAm-*co*-2St).

The overlap concentration, which Regalado et al.^33^ predict to be the critical aggregation concentration for hydrophobically modified PAAm, is slightly higher than 0.1 wt % (Figure 2). To test the DR capabilities at higher concentrations of (hydrophobically modified) polymers, we used a rotating “disc” apparatus (i.e., a rheometer) requiring lower solution volumes and thus less polymer. DR in Taylor–Couette flow differs from DR in turbulent pipe flow. Without polymer additive the instabilities in pipe flow and Taylor–Couette flow are inertia driven.^55^ Polymer addition introduces locally viscoelastic behavior which causes DR.^55^ In the presence of polymers Taylor–Couette flow in rotational geometry can be driven by elastic instabilities only.^59−61^ In pipe flow at a given flow rate, inertia-driven instabilities of a pure solvent are replaced by elasto-inertial instabilities in polymer solutions, resulting in drag reduction.^62,63^ In both inertia-elastic-driven turbulent viscoelastic pipe flow and elastically driven viscoelastic Taylor–Couette flow a cyclic drive^64^—velocity fluctuations generate stress fluctuations in polymer chains which produce velocity fluctuations when the elastic stress is released—is established. Polymer extension is induced by inertia in turbulent pipe flow, while elastic instabilities in rotational geometry are related to curved streamlines.^64^ Even though polymer molecules in Taylor–Couette flow might interact differently with the flow compared to turbulent pipe flow, it was shown that assessment of DR capabilities of polymeric drag-reducing agents is possible using a rotating “disk” apparatus,^18,44,45,45^ which might be attributed to the similarity in polymer–flow interaction. We performed these experiments in aqueous 0.025 M MgSO_4_ solution at concentrations up to 0.9 wt %. The DR results are presented as Prandtl–Kármán plots (Figure 6a–e). All tested polymers produced no DR at a concentration of 0.01 wt %. Also at 0.1 wt % no DR was observed. However, at concentrations of 0.35, 0.55, and 0.9 wt % the polymers displayed some DR capabilities (Table S3). While commercial PAAm having an *M*_*w*_ of 1000 kg/mol is more effective in reducing DR than the hydrophobically modified PAAm (1P(AAm-*co*-1St)), it must be noted that the hydrophobically modified PAAm started to foam during the experiments (Figure S10). Foaming started at a lower γ̇ threshold before the maximum DR was observed and increases with *c*. Foam formation resulted in an increased fluid volume in the test geometry, thus producing increased drag (see dotted lines in Figure 6c–e). Moreover, during foaming the effective polymer concentration in solution decreases significantly, which also affects its DR capabilities. Therefore, we were not able to quantify the real DR efficiencies of P(AAm-*co*-St) in the rotating “disc” apparatus.

The formation of very stable foams for hydrophobically modified PAAm was also encountered during the pipe flow experiments (Figure S11). The foam formation lowered the effective polymer concentration, thus resulting in lower DR. We air-dried the foam of 0.5P(AAm-*co*-2St) and obtained a solid polymer foam (Figure S11c). The foam volume as well as stability and density increased considerably for the copolymer with a higher hydrophobe content (0.5P(AAm-*co*-2St)) compared to the copolymer with a lower hydrophobe amount (1P(AAm-*co*-1St)) (Figure S11b and a, respectively). The reason for foam formation is still not fully understood, but a possible explanation is that the foam formed due to residual CTAB (present after purification) still being associated with the polymer. Assuming the concentration of CTAB in the copolymer sample to be 0.01 wt %, as shown by the elemental analysis results (Table S1), the total CTAB concentration in 300 L of polymer solution containing 30 g of polymer is 2.74 × 10^–8^ M, very much lower than the critical micelle concentration of CTAB (0.9 × 10^–3^ M), and thus insufficient to produce stable foams.^65^ However, CTAB copolymer complexes could act as polymeric surfactant due to their amphiphilic nature.

## Conclusion

Hydrophobically modified P(AAm-*co*-St) with different hydrophobe molar ratios and two *M*_*w*_ were synthesized using micellar copolymerization. The association properties of hydrophobically modified PAAm as well as unmodified reference PAAm in aqueous 0.025 M MgSO_4_ were determined using SLS and DLS. Their rheological and DR properties were compared to pure PAAm of similar *M*_*w*_. Our results provide evidence that hydrophobically modified copolymers produced using micellar polymerization with *M*_*w*_ ≈ 1000 kg/mol provided higher DR by a factor of ∼2 compared to the unmodified PAAm of similar *M*_*w*_ albeit at low DR level. DR increased with salt concentration, which promoted association formation. However, DR capabilities of copolymers with *M*_*w*_ < 1000 kg/mol were not improved in turbulent pipe flow compared to reference PAAm. Foam formation of modified polymers contributed to lowering the effective polymer *c*, resulting in decreased DR performance of the polymers. Even though associating polymers of lower *M*_*w*_ form high *M*_*w*_ associations, they seem to be easily destroyed already at low shear rates. In general, comparing *R*_η_ and *R*_g_/*R*_H_ the associations seem to be very sensitive to shear. In addition, DR measurements in the rotating “disc” apparatus showed no DR effect for polymer *c* < 0.1 wt %, while the DR effect in a horizontal pipe flow was observed already at *c* = 0.01 wt %. This observation highlights the essentiality of a combination of measuring techniques to adequately assess polymer-induced DR.

The concept of hydrophobically driven associations in aqueous solutions of modified PAAm improving long-term DR has to be still advanced to polymers forming more robust, higher association strength and larger associations to withstand higher shear rates.

## Supporting Information Available

Elemental analysis experimental procedure and results; supporting results such as ^1^H NMR spectrum, SLS and DLS graphs, additional rheological characterization, GPC spectra, DR graphs from ViEDRA measurements, photographs of foam formation (PDF)

## Symbols

*A*_2_: second virial coefficient (×10^–7^ mol dm^3^/g^2^)
*c*: concentration (wt %)
*c*_e_: entanglement concentration (wt %)
*c*_m_: mass concentration (g/mL)
*c**: overlap concentration (wt %)
*d*: diameter (mm)
*D*: translational diffusion coefficient (m^2^/s)
d*n*/d*c*: refractive index increment (mL/g)
DR: drag reduction (%)
*G*″: loss modulus (Pa)
*G*′: storage modulus (Pa)
*K*: Mark–Houwink parameter (mL/g)
*k*_B_: Boltzmann constant (m^2^ kg/s^2^ K)
*l*: length (m)
*L*: rotor height (mm)
*M*_η_: viscosity averaged molecular weight (kg/mol)
*M*_*w*_: weight-average molecular weight (kg/mol)
*n*: speed of rotation (s^–1^)
*N*_A_: Avogadro’s number (mol^–1^)
Δ*p*: pressure drop (Pa)
*R*_g_: radius of gyration (nm)
*R*_H_: hydrodynamic radius (nm)
*R*_*i*_: radii of the double-gap geometry (mm)
*R*_η_: viscosity averaged hydrodynamic radius (nm)
*R̅*: mean radius (mm)
*St*: amount of incorporated styrene in P(AAm-*co*-St) (mol %)
*T*: absolute temperature (K)
*u*: linear velocity (m/s)
*U*: volume averaged velocity (m/s)
*W*_M_: weight of the monomers (g)
*W*_P_: weight of the purified polymer (g)
*Y*: polymerization yield (%)

## Greek Letters

α: Mark–Houwink parameter
γ̇: shear rate (s^–1^)
γ̇_c_: critical shear rate at Taylor flow onset (s^–1^)
δ: ratio of the distance between rotor and stator of the double-gap geometry
η: apparent viscosity (Pa·s)
η_sp_: specific viscosity
η_r_: relative viscosity
[η]: intrinsic viscosity (mL/g)
η_0_: zero-shear viscosity (Pa·s)
ρ: solution density (kg/m^3^)
τ: shear stress (Pa)
φ: polymer volume fraction in solution (%)
ω: frequency (rad·s^–1^)
ω_c_: critical angular velocity at Taylor flow onset (rad·s^–1^)

## Dimensionless Groups

*Đ*: polydispersity
*f*: Fanning friction factor
*f*_p_: Fanning friction factor of polymer solution
*f*_s_: Fanning friction factor of pure solvent
*h̅*: average gap width
*I*_7.3 ppm_: integral corresponding to protons of phenyl groups (−C_6_H_5_)
*I*_1.5–1.8 ppm_: integral corresponding to protons of methylene groups (−CH_2_−)
*K*: geometrical factor
*Re*: Reynolds number

## Acronyms

AAm: acrylamide
Br: bromine
CTAB: hexadecyltrimethylammonium bromide
DLS: dynamic light scattering
GPC: gel permeation chromatography
HPLC: high-performance liquid chromatography
MWCO: molecular weight cutoff
NMR: nuclear magnetic resonance
PAAm: polyacrylamide
P(AAm-*co*-St): poly(acrylamide-*co*-styrene)
PEO: poly(ethylene oxide)
SLS: static light scattering
St: styrene
ViEDRA: Vienna Experiment for Drag Reducing Agents
