# Supplementary material for: Aqueous Solutions of Associating Poly(acrylamide-co-styrene): A Path to Improve Drag Reduction?
Source: Macromolecules. 2022 Nov 30;55(23):10479–90. doi: 10.1021/acs.macromol.2c01219 (PMC9753750; doi:10.1021/acs.macromol.2c01219)
Supplement: Supplementary file 1 — ma2c01219_si_001.pdf [file ma2c01219_si_001.pdf]

Supporting Information

Aqueous solutions of associating poly(acrylamide-co-styrene) - a path to improve drag reduction?

*Emina Muratspahić,[1,2] Lukas Brandfellner,[1,2] Jana Schöffmann,[1] Alexander Bismarck[1,3], Hans Werner Müller[1]**

[1]University of Vienna, Faculty of Chemistry, Institute of Materials Chemistry and Research, Polymer and Composite Engineering (PaCE) Group, Währinger Straße 42, 1090, Vienna, Austria

[2]University of Vienna, Doctoral College Advanced Functional Materials, Strudlhofgasse 4, 1090, Vienna, Austria

[3]Imperial College London, Department of Chemical Engineering, London SW7 2AZ, U.K.

* e-mail: hans.werner.mueller@univie.ac.at

EXPERIMENTAL SECTION

Elemental analysis. Elemental micro-analysis was performed at the faculty's Microanalysis Services department. Simultaneous determination of C, H, N and S was done by an automatic Elemental Analyser EA 3000 (Eurovector) using sample amounts between 1.0 and 1.5 mg. The instrument's operating procedure consists of a flash combustion combined with a Dumas-N-reduction and an on-line Gas Chromatography (GC) separation of the gaseous products N_2, CO_2, H_2O and SO_2. As a carrier gas high purity helium is used. A high temperature pyrolysis system HT 1500 (Hekatech) that operates at 1.480 °C was used for oxygen determination using similar sample amounts. Solid carbon acts as a reducing agent and high purity helium is used as a carrier gas. The analytical species detected is carbon monoxide. The instrument software CALLIDUS was used for data collection in both methods. Mineralisation of up to 5.0 mg of each sample in an AQF 100 (Mitsubishi) was performed to determine the halogene amount. Milli-Q-water was utilised as an absorbent for gaseous products. Using a 7100 CE (Agilent) and a conductivity detector (TraceDec) membrane filtration and capillary ion electrophoresis were employed to prepare and characterise the resulting solution. All instrument-data were evaluated using laboratory developed software (SCADA 5.99). A typical uncertainty for the results of main constituents is below 0.3 wt.%. The limit of quantification (LOQ) is 0.05 wt.% for N and 0.02 wt.% for S. Halogenes can be detected down to 0.01 wt.%.

SUPPLEMENTARY FIGURES AND TABLES

Table S1. Elemental composition of P(AAm-co-St) and the homopolymer obtaind using CHNS-O analysis

Sample	C (wt.%)	H (wt.%)	N (wt.%)	S (wt.%)	O (wt.%)	Br (wt.%)
Hompolymer	46.49 ± 0.01	7.23 ± 0.06	17.65 ± 0.05	< 0.02	26.67 ± 0.63	< 0.01
1P(AAm-co-1St)	46.55 ± 0.05	7.55 ± 0.05	17.62 ± 0.03	< 0.02	26.14 ± 0.30	< 0.01
0.7P(AAm-co-1St)	48.20 ± 0.88	7.35 ± 0.01	18.09 ± 0.32	< 0.02	25.26 ± 0.62	< 0.01
0.5P(AAm-co-2St)	46.42 ± 0.10	7.52 0.09	17.21 ± 0.06	< 0.02	26.18 ± 0.02	< 0.01

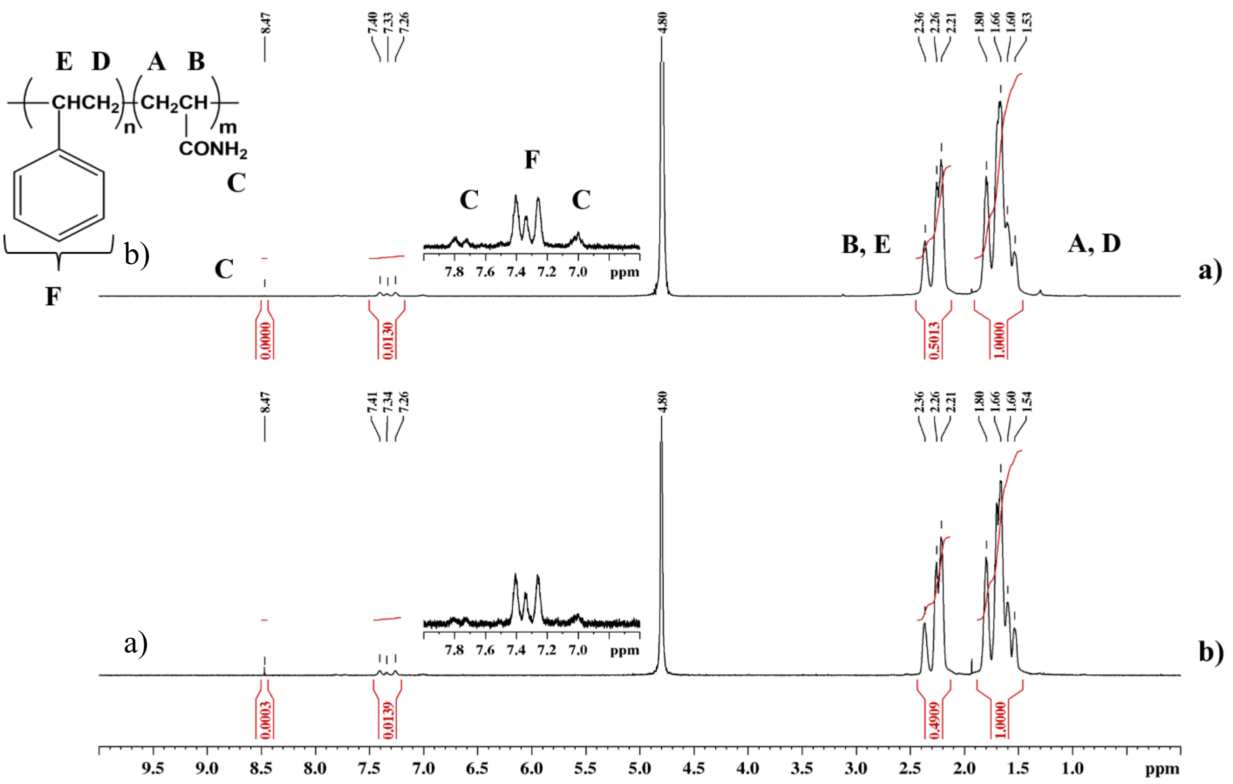

Figure S1. ^{1}HNMR spectrum of a) 0.7P(AAm-co-1St) and b) 1P(AAm-co-1St) acquired in D_2O

Light scattering

When analysing the SLS data the Zimm data reduction method provided negative intercept and consequently negative M_w for the 0.5P(AAm-co-2St) in aqueous $MgSO_4$. In fact, a reciprocal value for high M_w samples is very low and a little amount of signal noise can generate a negative extrapolated intercept.[1] We applied Berry's approach[2] which provides less curvature in the angular dependence for larger molecules. This way, linear extrapolation in the square-root of scattered light intensity $((Kc/R_\Theta)^{1/2}$ against angles and concentrations $q^2 + kc$ with k being a scaling factor yields a more reliable approximation. In order to ensure the consistency of the data, for all polymers acquired in aqueous $MgSO_4$ the data were processed by the same method (Figure S3). However, for the polymers analysed in formamide the Zimm method was applied since $1/M_w$ is sufficiently large (Figure S2).

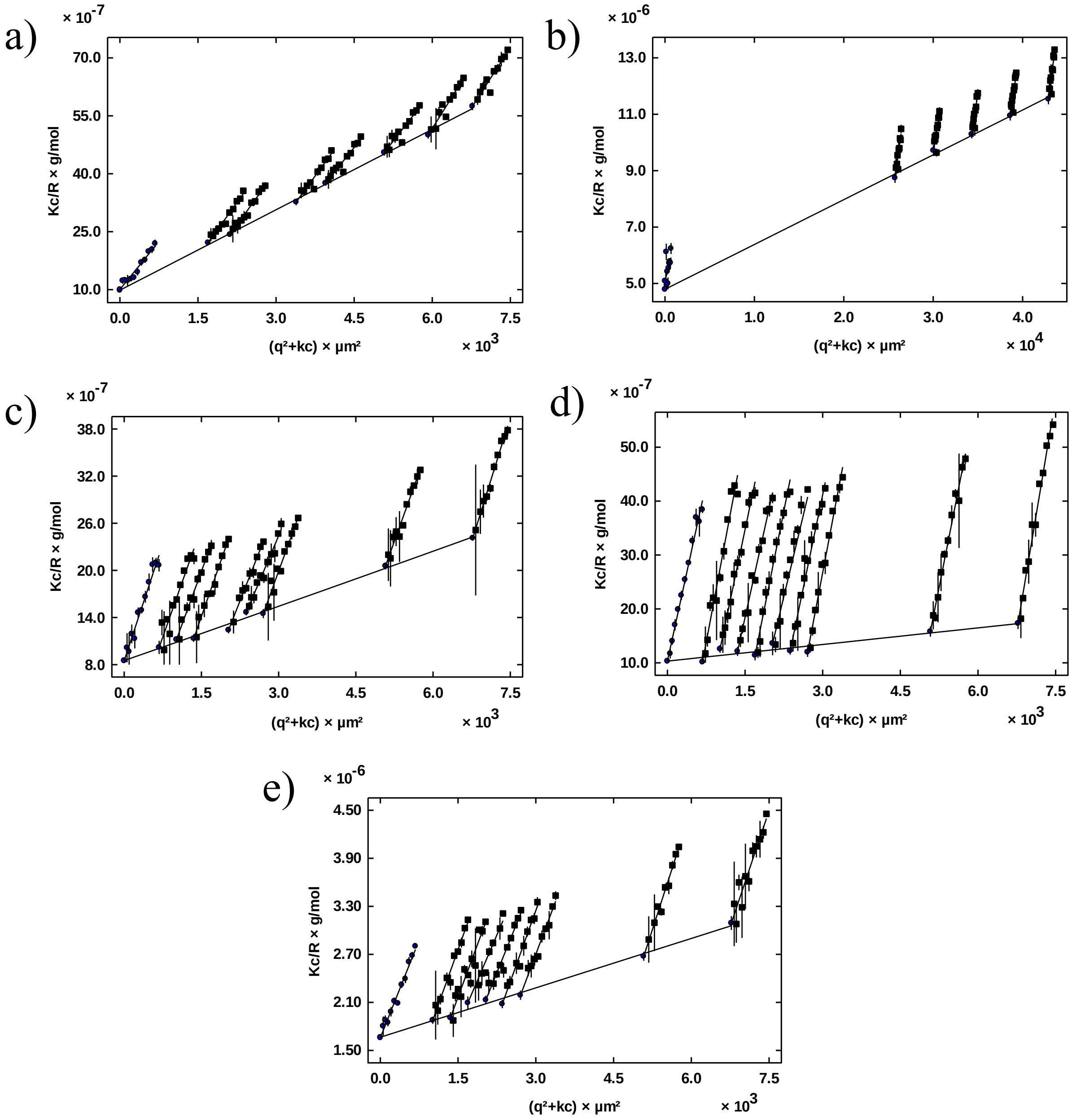

Figure S2. Static light scattering SLS data obtained in formamide and analysed using Zimm method for: a) PAAm1 b) PAAm0.5 c) 1P(AAm-co-1St), d) 0.7P(AAm-co-1St), and e) 0.5P(AAm-co-2St).

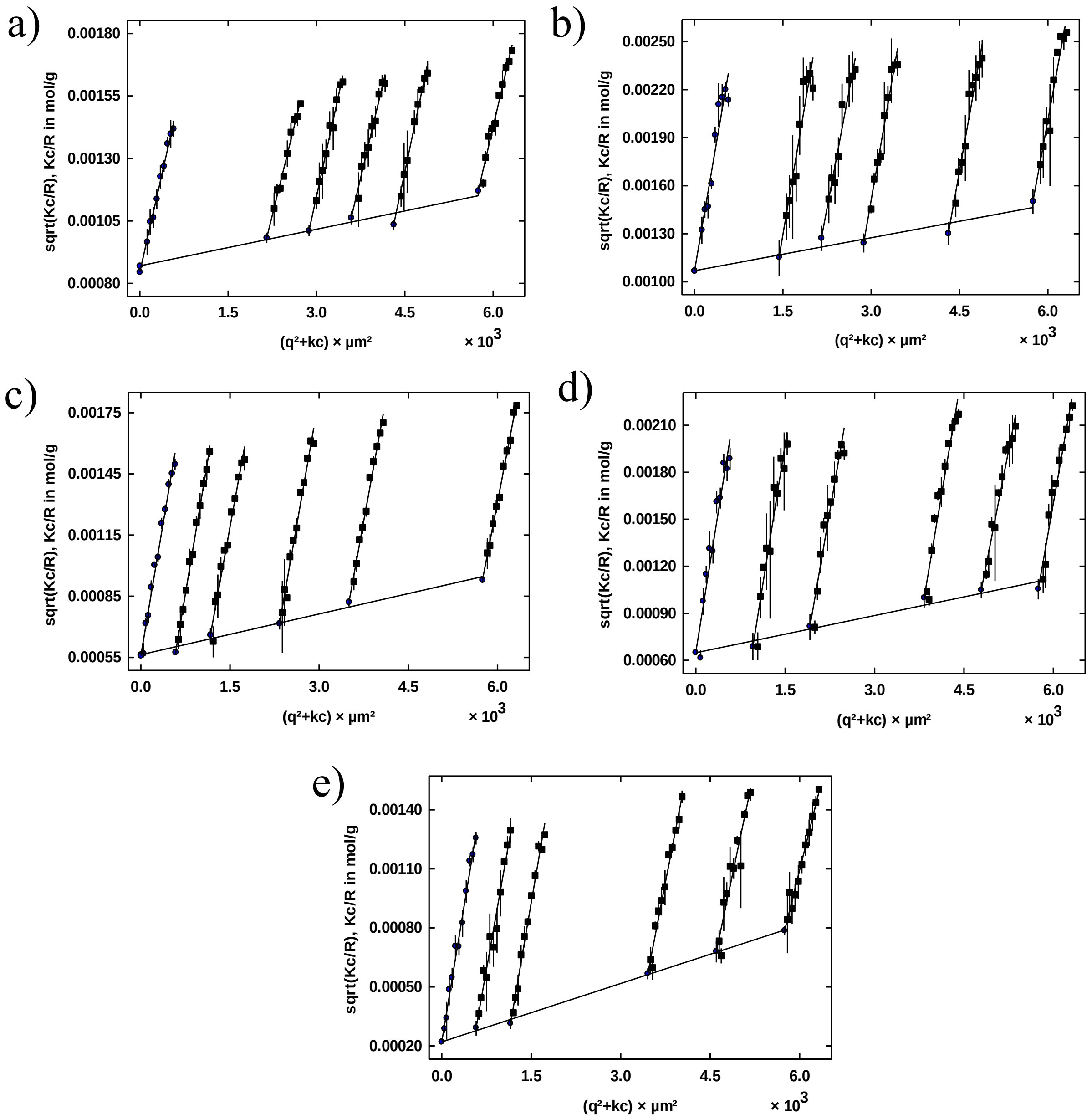

Figure S3. Static light scattering SLS data obtained in aqueous 0.025 M $MgSO_4$ and analysed using Berry method for: a) PAAm1 b) PAAm0.5 c) 1P(AAm-co-1St), d) 0.7P(AAm-co-1St), and e) 0.5P(AAm-co-2St).

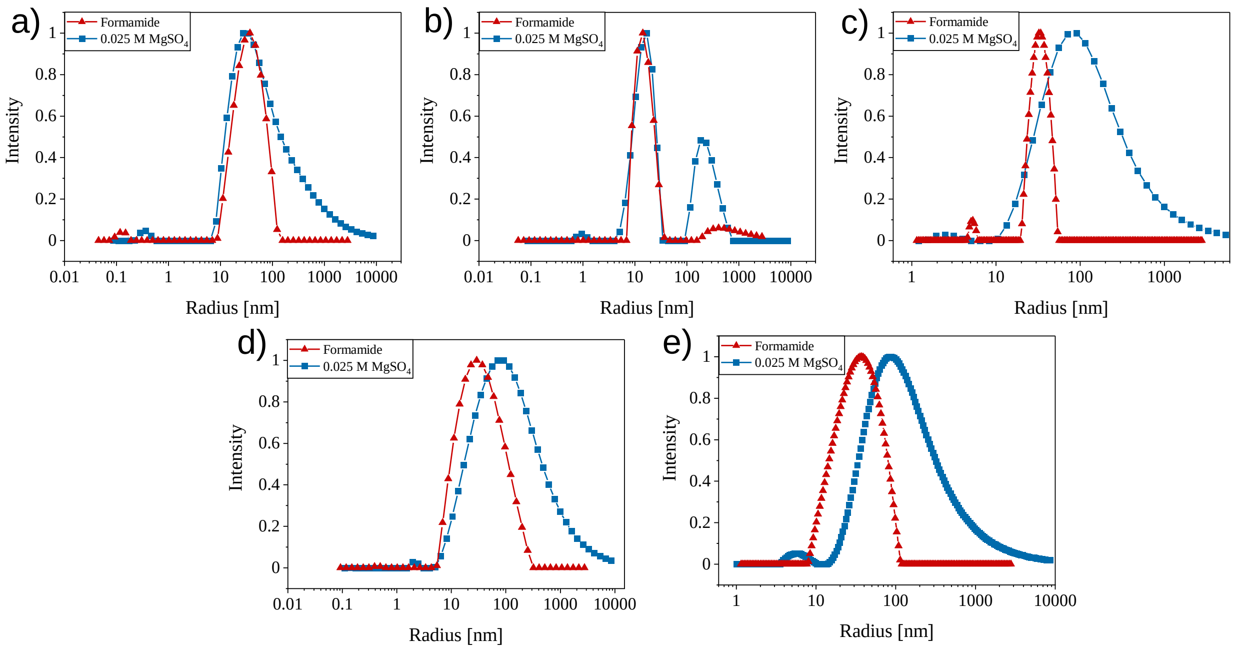

Figure S4. Dynamic light scattering DLS data obtained in formamide (red triangles) and aquoeus 0.025 M $MgSO_4$ (blue squares) for: a) PAAm1 b) PAAm0.5 c) 1P(AAm-co-1St), d) 0.7P(AAm-co-1St) and e) 0.5P(AAm-co-2St).

Zero shear viscosity

Table S2. Slopes of fit $ln(\eta_0) = \alpha \ln(c)$ for all polymers in three concentration regimes: dilute, semi-dilute unentangled and semi-dilute entangled regimes.

Sample	Dilute regime	Semi-dilute regime unentangled	Semi-dilute regime entangled
PAAm1	0.0343 ± 0.0069	1.0125 ± 0.1097	3.4549 ± 0.1111
1P(AAm-co-1St)	0.0727 ± 0.0122	0.9503 ± 0.0734	-
PAAm0.5	0.0132 ± 0.0021	0.4087 ± 0.0346	2.1416 ± 0.1927
0.7P(AAm-co-1St)	0.0231 ± 0.0037	0.7253 ± 0.0467	3.8178 ± 0.2364
0.5P(AAm-co-2St)	0.0181 ± 0.0035	0.6723 ± 0.0519	3.3978 ± 0.1029

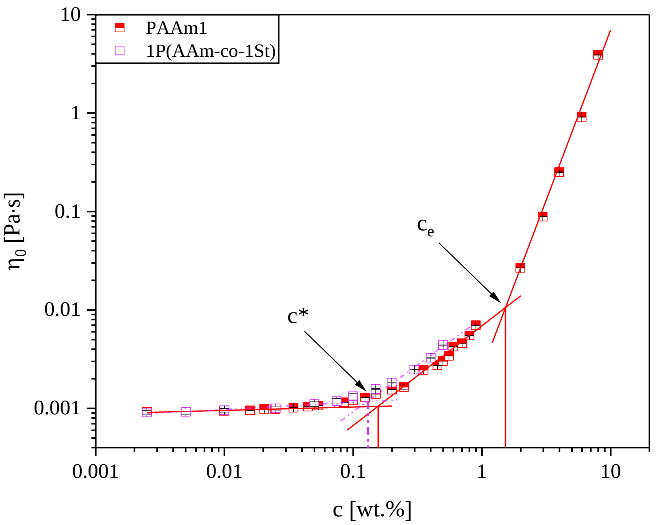

Figure S5. Concentration c dependence of the zero-shear viscosity η_0 determined in aqueous 0.025 M $MgSO_4$ for polymers with $M_w \approx 1000$ kg/mol.

Rheological characterisation in rheometer

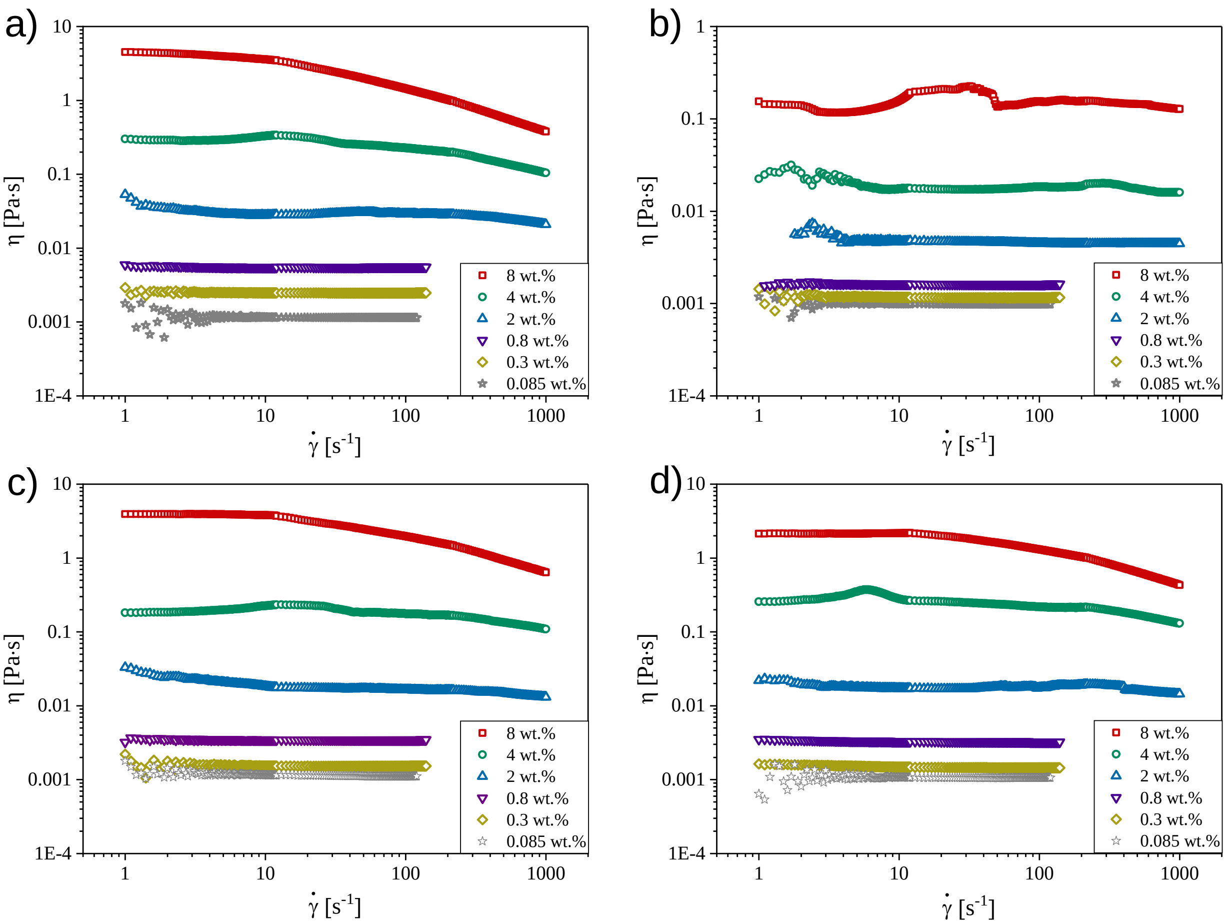

Figure S6. Shear rate $\dot{\gamma}$ dependence of viscosity η for: a) PAAm1, b) PAAm0.5, c) 0.7P(AAm-co-1St) and d) 0.5P(AAm-co-2St) obtained in aqueous 0.025 M $MgSO_4$ at different polymer concentrations.

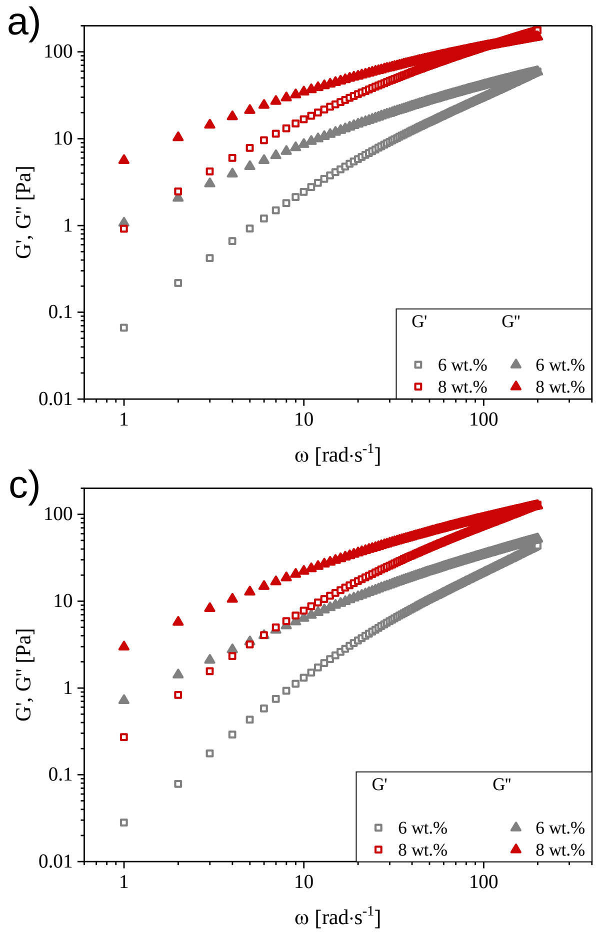

Figure S7. Frequency ω dependences of the storage (G') and loss (G'') moduli for: a) PAAm1, b) PAAm0.5, c) 0.7P(AAm-co-1St) and d) 0.5P(AAm-co-2St) obtained in aqueous 0.025 M $MgSO_4$ at polymer concentrations 6 and 8 wt.%.

Hydrophobically modified polymers aggregating into micellar structures at quiescent conditions undergo thermal fluctuations corresponding to approximately 1 $k_B T$.[3] The association network structure of end-functionalised telechelic polymers consists of "loops" formed by both polymer end groups being in a micelle, "dangling" chains having one of the ends without aggregation, and "bridging" chains that link two micelles.[3] In quiescent conditions the likelihood of hydrophobes associating into micelles is proportional to the probability of their detachment, while the attraction is significantly prominent when exposed to extensional deformation induced by increased

probability of the encounter of two hydrophobic ends.[3] Thus, telechelic polymers exhibit the steady shear viscosity profile that constitutes of a constant viscosity at low shear rates, shear thickening at intermediate shear rates, and pronounced shear thinning at high shear rates.[3] At low shear rates, thermal fluctuations govern the association processes.[3] The rates of aggregate formation and elongation-induced breakage rise nonlinearly as shear rates are enhanced.[3] This phenomenon first leads to increased number of "bridging" chains due to collision of hydrophobe ends (shear thickening), and later to lowered number of "bridging" chains caused by extensional deformation (shear thinning).[3] These shear and extensional deformation modes are in good agreement with the observations made by Sharma et al.[4] for multisticker associative polymers. Owing to concentration dependence of association processes, in dilute solutions very little number of "bridging" chains exists in the micelle network.[3] Martínez Narváez et al.[5] also examined the shear and extensional rheology response for associating hydrophobically modified hydroxyethyl cellulose ($M_w = 300$ kg/mol) and compared it to the unmodified hydroxyethyl cellulose ($M_w = 720$ kg/mol). The specific viscosity of the aqueous solutions of hydrophobically modified polymer was approximately the same as the one for the solutions of two times higher M_w of unmodified polymer.[5] Furthermore, a slight shear thickening in unentangled semidilute solutions of hydrophobically modified hydroxyethyl cellulose was observed, followed by more pronounced shear thinning when compared to the unmodified homopolymer.[5] These superior rheological properties of associative hydrophobically modified polymers are due to combination of associations and clustering.[5] The increased zero shear viscosity and relaxation time are caused by association−disassociation time and polymer chain-micelle dynamics.[5]

DR in turbulent pipe flow

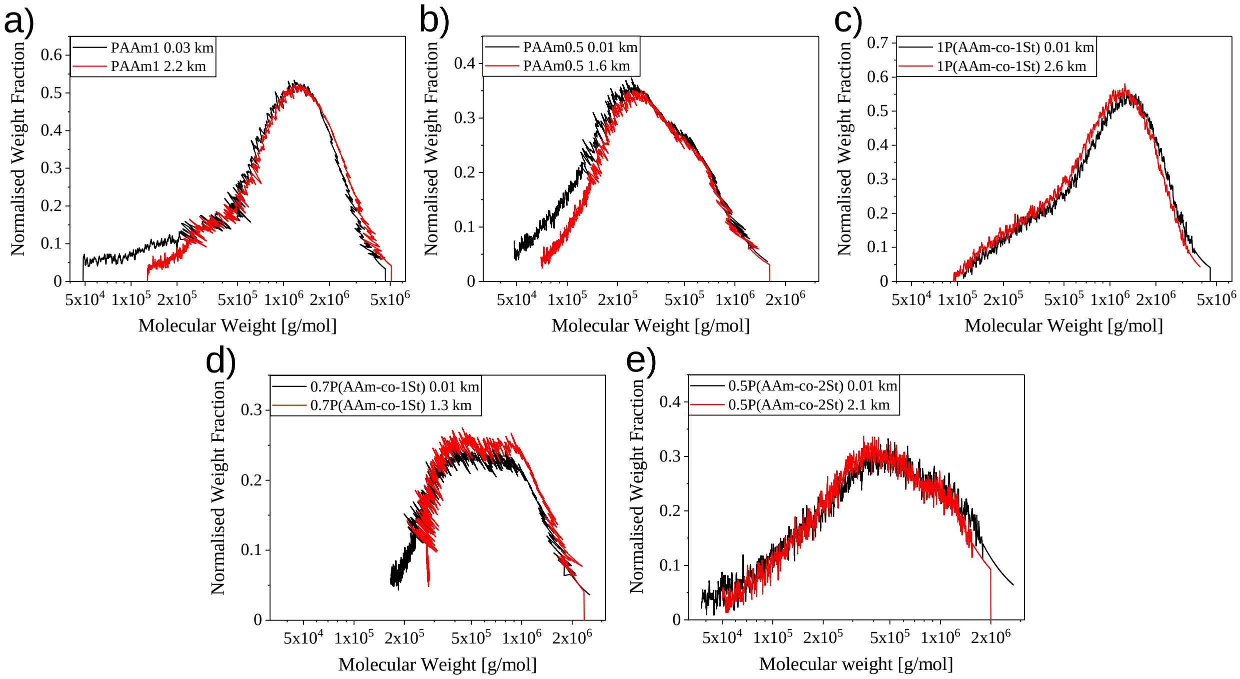

Figure S8. Molecular weight M_w distributions of the tested polymers after they travelled certain distances in ViEDRA: a) PAAm1, b) PAAm.05, c) 1P(AAm-co-1St), d) 0.7P(AAm-co-1St) and e) 0.5P(AAm-co-2St).

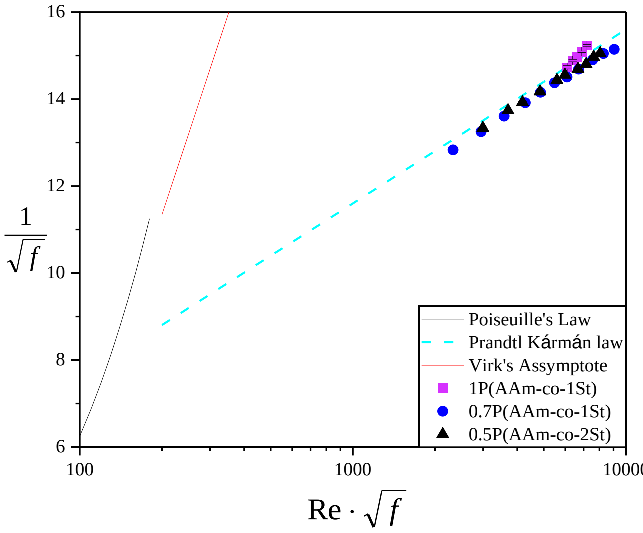

Figure S9. Prandtl-Kármán plot for hydrophobically modified polymers compared to Virk’s asymptote representing maximum DR achieved and Poiseuille’s law representing laminar flow.

DR experiments in rheometer

Drag reduction determined from the rheometer measurements at polymer $c > 0.1$ wt.%. Shown is the maximum DR that was achieved at the given experimental conditions and, in the case of hydrophobically modified polymers before excessive foaming started. These DR values were obtained at different Reynolds numbers Re. Re values for tested polymers were following: PAAm1 reached $Re_{\mathrm{c=0.35\ wt.\%}} = 980$, $Re_{\mathrm{c=0.55\ wt.\%}} = 700$, and $Re_{\mathrm{c=0.9\ wt.\%}} = 370$; 1P(AAm-co-1St) $Re_{\mathrm{c=0.35\ wt.\%}} = 1000$, $Re_{\mathrm{c=0.55\ wt.\%}} = 680$, $Re_{\mathrm{c=0.9\ wt.\%}} = 520$; PAAm0.5 $Re_{\mathrm{c=0.35\ wt.\%}} = 2000$, $Re_{\mathrm{c=0.55\ wt.\%}} = 1700$, $Re_{\mathrm{c=0.9\ wt.\%}} = 1300$; 0.7P(AAm-co-1St) $Re_{\mathrm{c=0.35\ wt.\%}} = 1100$, $Re_{\mathrm{c=0.55\ wt.\%}} = 690$, $Re_{\mathrm{c=0.9\ wt.\%}} = 390$; 0.5P(AAm-co-2St) $Re_{\mathrm{c=0.35\ wt.\%}} = 1100$, $Re_{\mathrm{c=0.55\ wt.\%}} = 700$, $Re_{\mathrm{c=0.9\ wt.\%}} = 400$. Beyond these Re excessive foaming or splashing occurred falsifying the measurement.

Table S3. Maximum drag reduction DR values obtained for tested polymers in the rheometer

Parameter	PAAm1	1P(AAm-co-1St)	PAAm0.5	0.7P(AAm-co-1St)	0.5P(AAm-co-2St)
$\mathrm{DR}_{\mathrm{c=0.35\ wt.\%}}$ (%)	17	7	0	5	4
Re	980	1000	2000	1100	1100
$\mathrm{DR}_{\mathrm{c=0.55\ wt.\%}}$ (%)	18	9	2	7	5
Re	700	680	1700	690	700
$\mathrm{DR}_{\mathrm{c=0.9\ wt.\%}}$ (%)	22	10	5	10	9
Re	370	520	1300	390	400

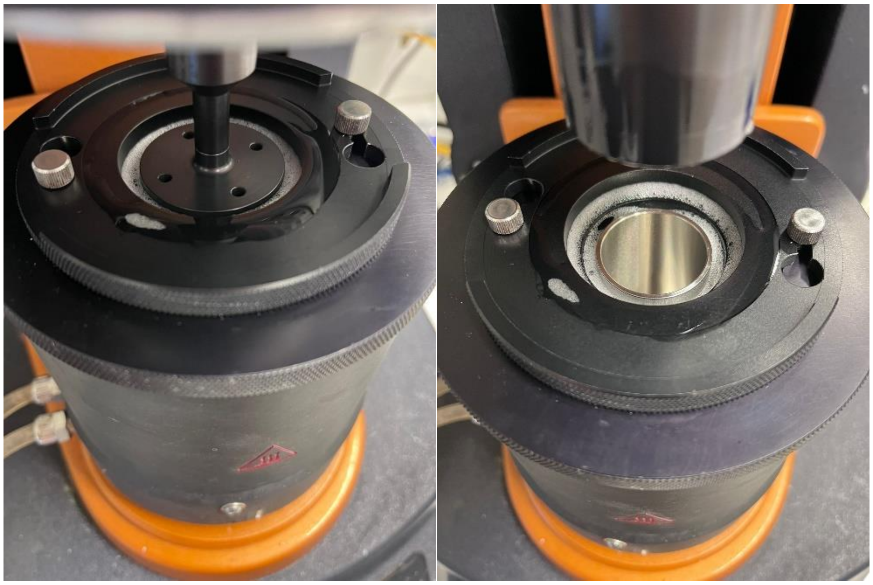

Figure S10. Foam formation for hydrophobically modified polymers during DR experiments in the rheometer.

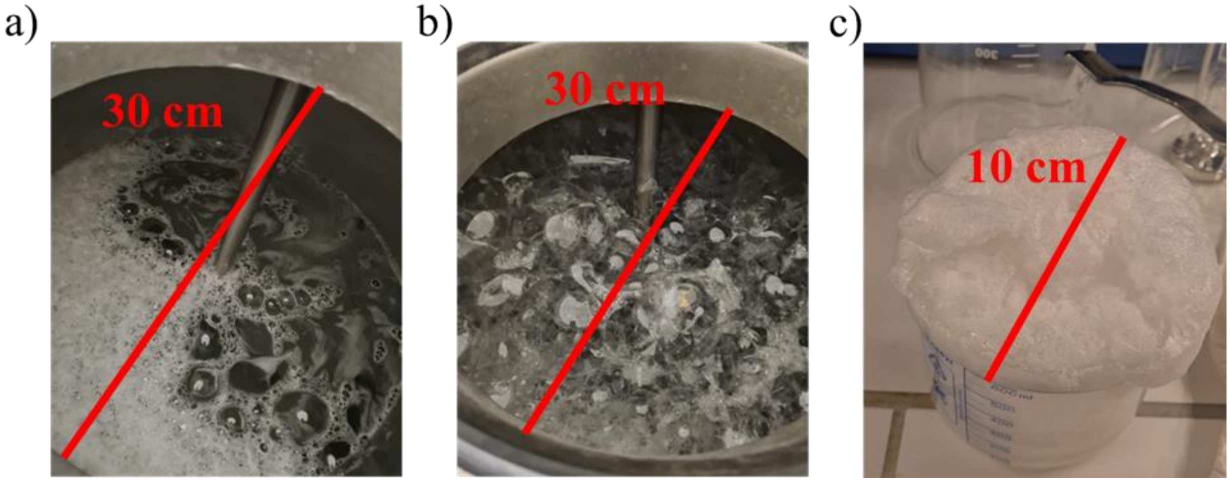

Figure S11. Foam obtained during DR experiments for hydrophobically modified polymers in ViEDRA: a) foam of 1P(AAm-co-1St) in mixng tank, b) foam of 0.5P(AAm-co-2St) in mixing tank, c) foam of 0.5P(AAm-co-2St) taken from the mixing tank and air dried

REFERENCES

1. Buchholz, B. A.; Barron, A. E. The Use of Light Scattering for Precise Characterization of Polymers for DNA Sequencing by Capillary Electrophoresis. *Electrophoresis* **2001,** *22* (19), 4118-4128.

2. Berry, G. Thermodynamic and Conformational Properties of Polystyrene. I. Light-Scattering Studies on Dilute Solutions of Linear Polystyrenes. *The Journal of Chemical Physics* **1966,** *44* (12), 4550-4564.

3. Tripathi, A.; Tam, K. C.; McKinley, G. H. Rheology and Dynamics of Associative Polymers in Shear and Extension: Theory and Experiments. *Macromolecules* **2006**, 39, 1981-1999.

4. Sharma, V.; Haward, S. J.; Serdy, J.; Keshavarz, B.; Soderlund, A.; Threlfall-Holmes, P.; McKinley, G. H. The Rheology of Aqueous Solutions of Ethyl Hydroxy-Ethyl Cellulose (EHEC) and its Hydrophobically Modified Analogue (hmEHEC): Extensional Flow Response in Capillary Break Up, Jetting (ROJER) and in a Cross-Slot Extensional Rheometer. *Soft Matter* **2015**, 11, 3251.

5. Martínez Narváez, C. D. V.; Dinic, J.; Lu, X.; Wang, C.; Rock, R.; Sun, H; Sharma, V. Rheology and Pinching Dynamics of Associative Polysaccharide Solutions. *Macromolecules* **2021**, 54, 6372−6388.
